# The calcium transient coupled to the L-type calcium current attenuates cardiac alternans

**DOI:** 10.3389/fphys.2024.1404886

**Published:** 2024-09-27

**Authors:** Mark Warren, Steven Poelzing

**Affiliations:** ^1^ Fralin Biomedical Research Institute at Virginia Tech Carilion, Roanoke, VA, United States; ^2^ Nora Eccles Harrison Cardiovascular Research and Training Institute, University of Utah, Salt Lake City, UT, United States; ^3^ Department of Bioengineering, University of Utah, Salt Lake City, UT, United States; ^4^ Department of Biomedical Engineering and Mechanics at Virginia Tech, Blacksburg, VA, United States; ^5^ Department of Internal Medicine at Virginia Tech Carilion, Roanoke, VA, United States

**Keywords:** alternans, action potential, calcium transient, L-type calcium current, ventricular myocytes

## Abstract

Cardiac action potential (AP) alternans have been linked to the development of arrhythmia. AP alternans may be driven by AP instabilities, Ca^2+^ transient (CaT) instabilities, or both. The mechanisms underlying CaT driven AP alternans is well-supported experimentally, but the ionic mechanism underlying alternans driven by AP instabilities remain incompletely understood. Here we used the Ca^2+^ buffer BAPTA to remove the CaT and generate a model of AP alternans driven primarily by AP instabilities. In isolated rabbit ventricle myocytes, AP alternans induced by rapid pacing were either critically damped and persisted over time, overdamped and ceased over seconds, or underdamped progressing to 2:1 capture. Control cells predominantly exhibited critically damped alternans. In contrast, removing CaT with BAPTA destabilized alternans formation in a concentration dependent manner. Importantly, alternans were easier to induce in CaT free cells as evidenced by a higher alternans threshold relative to control cells. While the L-type Ca^2+^ channel agonist Bay K 8644 had a minor effect on alternans formation in myocytes with conserved CaT, combining the agonist with BAPTA markedly promoted the formation of underdamped alternans and increased the alternans threshold more than four-fold as compared to controls. Our data support a mechanistic model in which AP alternans are a primary self-sustained event in which the CaT serves as a dampening cue that curbs alternans development, likely via a canonical negative feedback process involving Ca^2+^ induced inhibition of L-type Ca^2+^ current.

## 1 Introduction

Cardiac alternans have been linked to increased risk of sudden cardiac death, (Cutler and Rosenbaum, 2009; Verrier et al., 2011; Nearing and Verrier, 2002; Shusterman et al., 2006), and to the perpetuation of turbulent conduction during fibrillation (Karma, 1994; Garfinkel et al., 1997; Koller et al., 1998). While the cause of alternans is thought to be multifactorial (Qu and Weiss, 2023; Groenendaal et al., 2014; Pearman et al., 2018), two fundamentally distinct cellular mechanisms have been implicated in the generation of cardiac alternans. A first ‘voltage-driven’ mechanism involves beat-to-beat fluctuations in ion channel gating processes as the primary cause of action potential (AP) alternans (Kulkarni et al., 2019). In this scheme, time-dependent ion channel gating results in larger or smaller ionic currents every other beat, giving rise to the signature long-short-long-short pattern of AP alternans (Fox et al., 2002; Qu et al., 2010). Given this scenario, it has been suggested that the steepness of the AP duration (APD) restitution curve is a critical determinant of the development of voltage driven alternans, because from a mathematical standpoint a restitution curve slope of value one leads to the development of stable alternans (Nolasco and Dahlen, 1968; Karma, 1994; Shiferaw et al., 2005; Qu et al., 2010). Under the ‘voltage-driven’ paradigm, alternans in the intracellular calcium (Ca^2+^) transient (CaT) develop through an established sarcoplasmic reticulum graded Ca^2+^ release, whereby the magnitude of the triggered CaT is proportional to local Ca^2+^ entry via L-type calcium channels (I_CaL_) (Beuckelmann and Wier, 1988; Callewaert, 1992; Cannell et al., 1987). A second ‘Ca^2+^-driven’ mechanism involves beat-to-beat fluctuations in the sarcoplasmic reticulum Ca^2+^ release and refilling dynamics that elicit an large-small-large-small type pattern (alternans) in the CaT as a primary event. CaT alternans are then transmitted to the transmembrane potential oscillations via bidirectional coupling existing between the voltage and Ca^2+^ signals (Kulkarni et al., 2019; Qu and Weiss, 2023).

To date, the cellular mechanisms of ‘Ca^2+^-driven’ alternans have been extensively studied (Qu and Weiss, 2023), but the ionic mechanisms underlying the development of ‘voltage driven’ type alternans remain incompletely understood. Previously, simulation studies suggested that alterations in CaT induced inactivation of I_CaL_ and the current’s effect on APD, can shift the alternans threshold (Fox et al., 2002). To experimentally test this hypothesis, we buffered intracellular Ca^2+^ cycling using BAPTA to effectively uncouple AP alternans from the CaT. Given that the strong buffering effect of 10 mM BAPTA, a standard concentration used in various studies, may not be sufficient to completely remove CaT induced inactivation of I_CaL_ (Dick et al., 2008), we compared the effect of 10 mM BAPTA to that of 20 mM BAPTA. Since CaT induced inactivation of I_CaL_ was shown to be BAPTA concentration dependent (Sham, 1997), and since 20 mM BAPTA was shown to prominently reduce CaT induced inactivation of I_CaL_ (Zong and Hofmann, 1996), we expected that comparison of 10 mM and 20 mM BAPTA would yield an insight into how local Ca^2+^ signaling may modulate alternans formation. We then investigated how agonizing I_CaL_ modulated the formation of AP alternans in cells with either intact or buffered intracellular Ca^2+^ cycling. Our data supports computational predictions that the CaT serves as a dampening cue that inhibits the development of AP alternans via I_CaL_. Interruption of the dampening cue markedly modifies the threshold of alternans onset and the dynamic patterns of AP alternans in a manner which depends on BAPTA concentration. When intracellular Ca^2+^ buffering and agonizing I_CaL_ are combined, the formation of unrestrained (aka under-damped) alternans is promoted. The data support a paradigm in which the interaction between AP and CaT during the formation of alternans is best described by a negative feedback loop in which the CaT serves as the dampening cue. The results may have significant implications in the translational management of cardiac alternans.

## 2 Materials and methods

### 2.1 Study design

The objective of the study was to investigate the incidence, characteristics, and ionic mechanism of AP alternans developing in an experimental model in which we eliminated the CaT, a Ca^2+^ signal that has been proposed to be the source of AP alternans. All procedures were approved by the Animal Care and Use Committee of the University of Utah and complied with the American Physiological Society’s *Guiding Principles in the Care and Use of Animals.* The experiments were carried out using isolated rabbit (male and female) ventricular myocytes subject to continued pacing at progressively faster rates to promote the development of AP alternans. Myocytes were superfused with a physiological solution which was untreated (group I, Control) or treated with a I_CaL_ agonist (group II) to determine the effect of stimulating I_CaL_ on the formation of alternans in cells with conserved intracellular Ca^2+^ cycling. Myocytes were also treated with a Ca^2+^ buffer loaded into the intracellular compartment to determine the effect of buffering the CaT on the formation of AP alternans (groups III and IV, each at a distinct concentration of the buffer), and to determine how buffering CaT interacted with a I_CaL_ agonist. In the latter condition, the unexpected effect of combining the two test drugs prompted a pacing protocol modification such that rather than subjecting the cells to progressive increases in the pacing rate, we had to reduce the pacing rate applied to the cell progressively in order achieve a pacing response of one captured beat for every stimulus. Pacing induced AP responses were recorded continuously using the patch clamp technique and intracellular Ca^2+^ changes measured in tandem for brief intervals (∼2s) during pacing by means of the Ca^2+^ sensitive fluorescent probe fluo-4. Provided that paced myocytes did not always develop alternans, the total number of cells per group was adjusted in order to obtain 8–14 alternans positive cases in each experimental condition. The investigators were not blinded to the experimental interventions. However, experimental protocols and data analysis were designed to include all myocytes (i.e., alternans positive and alternans free), and an offline computationally driven data analysis implemented so as to remove bias in the quantification of AP alternans by selecting an objectively predetermined section of the AP recordings for analysis, which was equivalent for all experimental conditions. For each myocyte, computational analysis of each AP in response to the complete pacing protocol was implemented via custom software, and used to identify AP alternans sequences.

### 2.2 Myocyte isolation and electrophysiological methods

Adult New-Zealand White rabbit (1.5–1.7 Kg) ventricular myocytes were isolated by combining enzymatic tissue digestion and mechanical trituration (supplement for details) (Saegusa et al., 2013). Transmembrane voltage (V_m_) was continuously recorded using a glass pipette (2–4 MΩ tip) and an Axoclamp 2B amplifier (Molecular Devices, Ca) in bridge mode (supplement for details) (Zaniboni et al., 2000). To elicit an AP, we injected a rectangular depolarizing current pulse (duration 3–4 ms) into the myocytes through the patch pipette (supplement for details). Using a previously described approach we loaded the fluorescent probe fluo-4 into the isolated myocytes to track the changes in intracellular Ca^2+^ (Saegusa et al., 2013; Saegusa et al., 2011). Fluo-4 fluorescence signals were recorded using an EMCCD camera (iXon 860, Andor Technology, Belfast, United Kingdom) configured to record images at a resolution of 64 × 64 pixels and 860 frames/s (supplement for details) (Warren et al., 2010). Simultaneously recorded APs and CaTs were aligned for analysis (supplement for details) (Supplementary Figure S1). Where indicated, myocytes were loaded through the glass pipette with 10.0 mM or 20.0 mM BAPTA [1,2-Bis(2-aminophenoxy)ethane-N,N,N′,N′-tetraacetic acid; Tocris Bioscience, United Kingdom)] (Saegusa et al., 2013), a Ca^2+^ buffer used to effectively reduce the CaT beyond detection. Experiments were carried out at 36.5 ± 1.0°C using Rod-shaped myocytes with well-defined striations.

Once a myocyte was attached to the patch pipette, stimulus pulses were delivered at a pacing cycle length (PCL) of 2000 (or 1000) ms for 2–7 min to establish steady state conditions. Therein, the cells were subject to progressively shorter PCL with the objective of inducing AP alternans (Supplementary Figure S2A). The target PCLs were (in ms): 2000, 1000, 500, 300, 180. Given that each cell responded uniquely to changes in rate, a number of cells were subject to intermediate PCL during implementation of the pacing protocol. At each PCL (including intermediate rates) the cells were constantly paced for ∼30–∼90 s to allow for adaptation before shortening the PCL (Supplementary Figure S2A). Ultimately, the PCL was reduced to attain a final value of 180 ms unless the development of alternans occurred before.

### 2.3 Experimental protocols

The pacing protocol (Supplementary Figure S2A) was applied to myocytes from four experimental groups: I) myocytes patched with normal pipette solution and superfused with normal Tyrode solution, *Control group* (n = 22); II) cells patched with normal pipette solution and superfused with Tyrode’s solution containing 25 nM Bay K 8644, *Bay K 8644 group* (n = 9); III) cells patched with pipette solution containing 10 mM BAPTA and superfused with normal Tyrode solution, *10 mM BAPTA group* (n = 10); IV) cells patched with pipette solution containing 20 mM BAPTA and superfused with normal Tyrode solution, *20 mM BAPTA group* (n = 9). In the Bay K 8644 group, the pacing protocol was initiated once delivery of Bay K 8644 at baseline elicited a visible effect on the AP (i.e., AP prolongation).

To test the effect of Bay K 8644 on myocytes lacking CaT, myocytes loaded with 10 mM BAPTA bathed in control solution were exposed to 25 nM Bay K 8644 during constant pacing at 500–1000 ms PCL (n = 8). The PCL was adjusted to ensure 1:1 capture at the fastest possible rate as required by the drug effects.

### 2.4 Detection of APD alternans

Data reported were generated from the last 40 activations recorded at each PCL (Supplementary Figure S2A). For each AP in this ‘analysis window’ we determined the APD at 90% repolarization (APD_90_) and the duration of the preceding diastolic interval (DI) (supplement for details). Representative AP’s and APD_90_ sequences during long-short-long-short type responses are shown in Supplementary Figure S2B (upper and lower, respectively). For each 40 activation-long ‘analysis window’ we determined the maximum number of consecutive APD_90_ pairs locked in a long-short-long-short type pattern, which we will also refer to as 2:2 type response/pattern. If seven or more consecutive APD_90_ pairs exhibiting 2:2 type responses were identified we designated the myocytes as ‘alternans positive’, following the probabilistic reasoning introduced by Dilly and Lab (1988) The longest applied PCL satisfying the alternans positive criteria was defined as the ‘alternans threshold’ (ALT-TH). Myocytes devoid of APD_90_ sequences satisfying the ‘alternans positive’ criteria were classified as ‘alternans free’. Activation in alternans free sequences is also referred to as 1:1 type response.

### 2.5 Data analysis and statistical tests

Data are presented as mean ± standard deviation. For each PCL applied to a given myocyte, we computed the mean DI (Mean-DI) and mean APD_90_ (Mean-APD_90_) as the average DI and APD_90_ values of activations conforming the 40-activation-long analysis window (Supplementary Figure S2A). We then plotted Mean-APD_90_ *versus* Mean-DI to generate an APD restitution curve for each myocyte. Myocytes where classified into either an ‘alternans positive” or ‘alternans free’ category, and the segregated data used for group-wise and/or category-wise statistical analysis by means of the two-sided Student’s t-test. We used the Chi-squared test to compare the group-wise fraction of alternans positive myocytes and the group-wise distribution of alternans types. For each 40 activation-long activation sequences meeting the ‘alternans positive’ criteria we determined the alternans magnitude (ALT-MAG) as follows. Initially we identified each APD_90_ pair locked in a long-short-long-short type pattern. For each pair we then calculated the difference in APD_90_ values of the first AP in the pair minus the second AP. Finally, by averaging the absolute values of these differences over all pairs exhibiting the 2:2 type response, we determined ALT-MAG. To determine the slope of the APD_90_ restitution curve at the ALT-TH we determined the slope of the linear function connecting two specific data points of the APD_90_ restitution curve: data-point#1 consisted of the Mean-DI/Mean-APD_90_ pair corresponding to the ALT-TH, and data-point#2 consisted of the Mean-DI/Mean-APD_90_ pair with next largest Mean-DI value. Statistical comparisons yielding *p* values less than 0.05 were considered significant.

## 3 Results

### 3.1 Incidence of AP alternans

**FIGURE 1:**
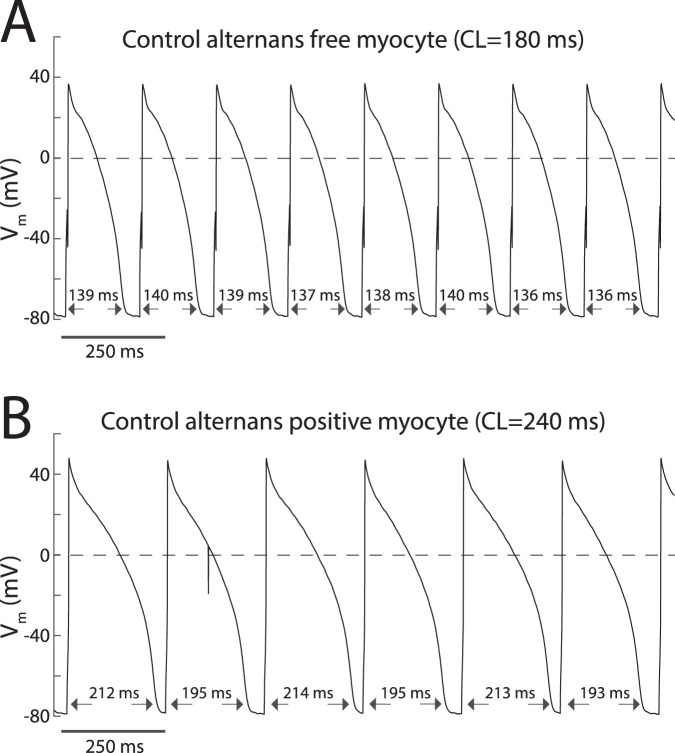
Incidence of AP alternans in myocytes is heterogeneous. **(A)** Transmembrane potential recordings acquired from an alternans free myocyte depicting a sequence of APs for which APD_90_ values fluctuate stochastically. **(B)** Transmembrane potential recordings acquired from an alternans positive myocyte depicting a sequence of APs for which APD_90_ values exhibit a 2:2 type patern.

Action potential (AP) alternans could be induced by rapid pacing in some but not all myocytes. Figure 1 depicts representative transmembrane potential changes recorded from control ‘alternans free’ (Figure 1A) and ‘alternans positive’ (Figure 1B) myocytes. Note that the AP sequence of the ‘alternans positive’ myocyte exhibits a long-short-long-short APD_90_ pattern (Figure 1B), whereas the ‘alternans free’ does not (Figure 1A). Group-wise analysis showed that there were no significant differences in the fraction of ‘alternans positive’ cells between control cells (14/22) and cells perfused with the I_Ca,L_ agonist Bay K 8644 at 25 nM concentration (8/9; *p* = 0.16), or cells loaded with either 10 mM BAPTA (7/10; *p* = 0.73 vs. control) or 20 mM BAPTA (8/9; *p* = 0.16 vs. control) to buffer CaT.

### 3.2 AP and CaT alternans

**FIGURE 2:**
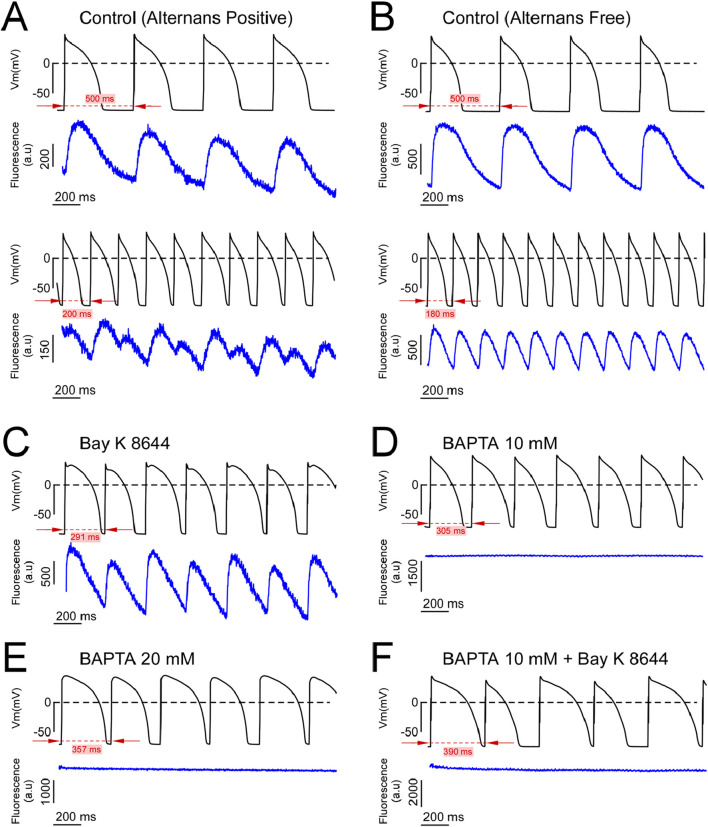
Dual AP-CaT recordings from alternans positive and alternans free cells. **(A, B)** Simultaneous AP (upper trace, black) and fluo-4 fluorescence (lower trace, blue) recordings acquired during slow (upper pair of traces) and rapid (lower pair of traces) pacing from representative control alternans positive **(A)** and alternans negative **(B)** myocytes. **(C–F)** Simultaneous AP (upper trace, black) and fluo-4 fluorescence signals (lower trace, blue) recorded at (or below) the alternans threshold of alternans positive myocytes from the Bay K 8644 **(C)**, 10 mM BAPTA **(D)**, 20 mM BAPTA **(E)**, and 10 mM BAPTA plus Bay K 8644 **(F)** groups.

It has been hypothesized that CaT alternans drive AP alternans (Qu and Weiss, 2023). Therefore, to determine the necessity of CaT in the formation of AP alternans in our model of ‘free-running’ AP alternans developing at physiological temperature, we recorded AP with current clamp and CaT with optical methods simultaneously in alternans free and alternans positive sequences for all experimental conditions. Figure 2A depicts simultaneous AP and CaT signals acquired during PCLs above (upper pair) or below (lower pair) the alternans threshold (ALT-TH) from an alternans positive Control myocyte. Specifically, note how shortening the PCL from 500 ms to 200 ms shifted the response of both the AP and the CaT signals from a non-alternans (1:1) to an alternans (2:2) type response. Due to the finite and relatively short optical CaT recordings relative to continuous patch clamp recordings, we could not determine if either the AP or CaT alternans preceded one another, or if alternans developed simultaneously in both signals. All dual AP and CaT recordings acquired during the development of AP alternans (12 dual recordings from eight different cells) revealed an in-phase 2:2 capture in the AP and CaT signals, such that long APD was accompanied by large amplitude CaT, and conversely short APD with a small amplitude CaT (Figure 2A, lower two traces). Simultaneous AP and CaT recordings from a representative alternans free Control myocyte depict the absence of AP alternans in the 500–180 ms range of PCLs (Figure 2B). Note that the simultaneously recorded CaT signals were also alternans free (Figure 2B, blue traces).

In myocytes perfused with the I_Ca,L_ agonist Bay K 8644, both AP and CaT developed in-phase alternans (Figure 2C). Importantly, intracellular loading of either 10 or 20 mM of the Ca^2+^ chelator BAPTA reduced the CaT below detection, yet AP alternans were readily inducible (Figures 2D, E, respectively). Finally, the addition of Bay K8644 to agonize I_Ca,L_ did not restore the CaT buffered by 10 mM BAPTA, yet AP alternans were still inducible (Figure 2F). These data demonstrate that AP and CaT can alternate in phase, and AP alternans can occur in the absence of a measurable CaT.

### 3.3 AP alternans characteristics

**FIGURE 3:**
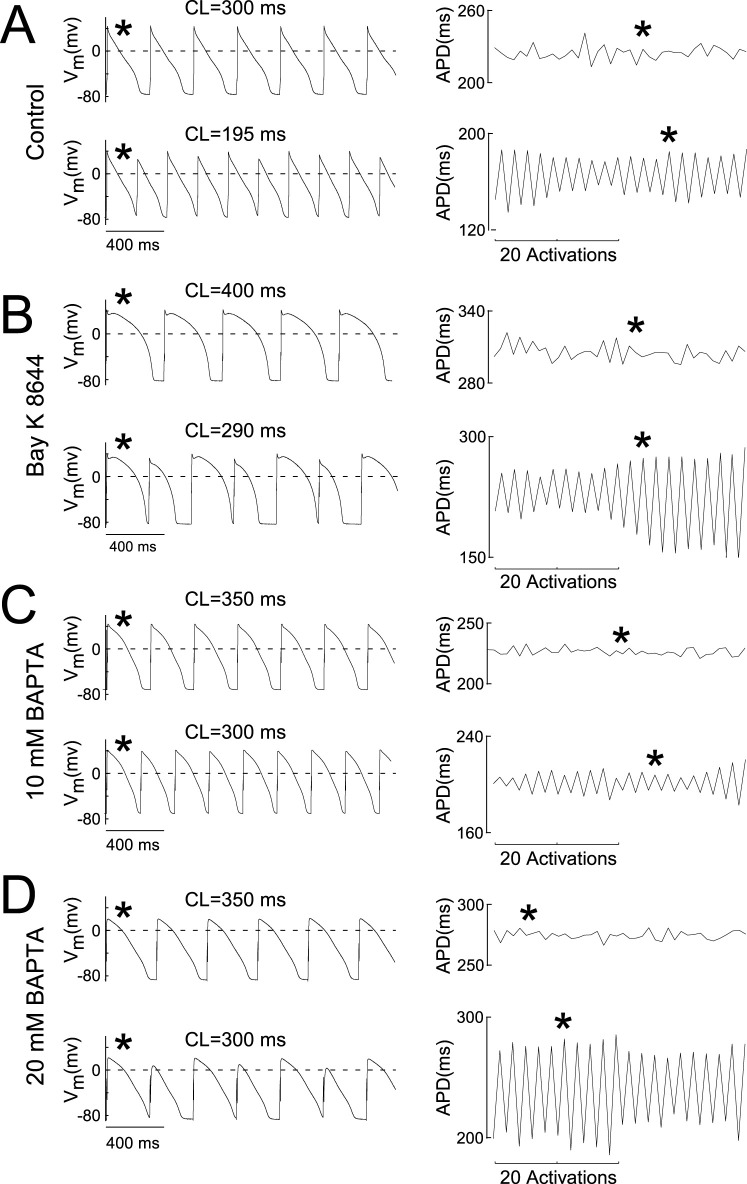
AP alternans prevail in Ca^2+^ buffered cells. **(A–D)** Leftmost pairs of traces depict APs recorded during constant pacing before the onset of alternans (upper trace) and at the alternans threshold (lower trace depicting alternans) in control myocytes **(A)**, myocytes superfused with 25 uM Bay K 8,644 **(B)**, myocytes loaded with 10 mM BAPTA **(C)**, and myocytes loaded with 20 mM BAPTA **(D)**. Rightmost pairs of traces in each panel depict the 40 activation-long APD_90_ sequences used to evaluate the presence of alternans at the corresponding PCL. The asterisk indicates the correspondence between the AP and the APD_90_ measure. Labels above the AP recordings indicate the PCL.

Representative AP recordings and the corresponding APD_90_ over time for two PCLs are presented in Figure 3 for untreated Control cells (Figure 3A, leftmost and rightmost panels, respectively), cells treated with Bay K 8466 (Figure 3B, leftmost and rightmost panels, respectively), and cells treated with BAPTA (Figures 3C, D, leftmost and rightmost panels, respectively). For each experimental condition, transmembrane potential recordings were acquired during pacing at a PCLs above the ALT-TH (upper trace, leftmost panels) or equal to or below the ALT-TH (lower trace, leftmost panels). Importantly, the data demonstrate the ability to measure an uninterrupted 2:2 type pattern of APD_90_ alternans lasting at least 40 activations for all conditions (Figures 3A–D lower trace, rightmost panels).

To determine the relationship between the transmembrane potential properties and the development of AP alternans in the various experimental groups, we tabulated and compared the average values of the Mean-DI and of the Mean-APD_90_ at a PCL of 500 ms, a rate close to the intrinsic heart rate of rabbits (normocardia) and above the ALT-TH. Summary data in Table 1 shows that Mean-APD_90_ at 500 ms PCL (normocardia) was 252 ± 53 ms in controls and similar in cells treated with Bay K 8466 (296 ± 65 ms, *p* = 0.059) (Table 1). Note, an effect of Bay K 8466 was observed as evidenced by Mean APD_90_ prolongation from 270 ± 107 ms to 323 ± 116 ms (*p* < 0.0001) during bradycardic pacing (PCL = 1000 ms). Both 10 mM BAPTA and 20 mM BAPTA increased normocardic Mean-APD_90_ to 367 ± 56 ms and 337 ± 43 ms respectively (*p* < 0.0005, Table 1).

**TABLE 1:** AP alternans characteristics.

Group	PCL = 500 ms		Group	PCL = ALT-TH				
	Mean-DI (ms)	Mean-APD_90_ (ms)		ALT-TH (ms)	Mean-DI (ms)	Mean-APD_90_ (ms)	Number 2:2 A P’s (count)	ALT-MAG (ms)
Control (n = 22)	248 ± 51	252 ± 53	Control (n = 14)	229 ± 36	47 ± 17	182 ± 34	28 ± 10	17 ± 15
Bay K 8644 (n = 9)	201 ± 65^a^	296 ± 65	Bay K 8644 (n = 8)	262 ± 72	56 ± 14	206 ± 60	27 ± 11	27 ± 36
10 mM BAPTA (n = 10)	130 ± 55^b^	367 ± 56^b^	10 mM BAPTA (n = 7)	288 ± 91^a^	49 ± 13	240 ± 79^a^	24 ± 9	13 ± 6
20 mM BAPTA (n = 9)	160 ± 43^b^	337 ± 43^b^	20 mM BAPTA (n = 8)	284 ± 50^b^	53 ± 11	232 ± 44^b^	24 ± 11	20 ± 22

^a^ *p* < 0.01, Student’s t-test, compared to control values of the same PCL, category.

^b^ *p* < 0.0005, Student’s t-test, compared to control values of the same PCL, category.

AP, action potential; ALT-TH, alternans threshold; ALT-MAG, alternans magnitude; APD90, Action Potential Duration at 90% repolarization; DI, diastolic interval; PCL, pacing cycle length.

We next quantified the ALT-TH as previously described (Pruvot et al., 2004). In Controls, the ALT-TH was 229 ± 36 ms (Table 1). Consistent with the lack of effect of Bay K 8466 on normocardic Mean-APD_90_, the I_CaL_ agonist did not significantly increase the ALT-TH relative to Controls (262 ± 72 ms, *p* = 0.16) (Table 1). In contrast, AP alternans were induced at longer PCLs in the absence of the CaT. Specifically, the average ALT-TH in cells perfused with 10 mM BAPTA and 20 mM BAPTA were both significantly greater than the ALT-TH for Controls (288 ± 91 ms and 284 ± 50 ms; *p* = 0.045 and *p* = 0.0065 respectively) (Table 1).

At the ALT-TH, Mean-APD_90_ was yet again not significantly different between Control (182 ± 34 ms) and cells perfused with Bay K 8466 (206 ± 60 ms, *p* = 0.24) (Table 1). In contrast, the average Mean-APD_90_ at the ALT-TH was significantly increased by 10 mM BAPTA (240 ± 79 ms, *p* = 0.029) and 20 mM BAPTA (232 ± 44 ms, *p* = 0.007), consistent with the significantly greater ALT-TH observed in both CaT buffered groups (Table 1).

To further assess the nature of the AP instabilities developing at the ALT-TH, we quantified the number of consecutive APD_90_ alternans pairs developing in the 40 activation-long window corresponding to the ALT-TH. Of note, we did not detect a significant difference between the number of consecutive 2:2 AP’s identified in Controls and cells treated with 25 nM Bay K 8466 (*p* = 0.99), 10 mM BAPTA (*p* = 0.42), or 20 mM BAPTA (*p* = 0.42) (Table 1). Additionally, the alternans magnitude (ALT-MAG) measured at the ALT-TH did not exhibit group-wise differences (*p* = 0.45 for Control vs. Bay K 8644; *p* = 0.77 for Control vs. 10 mM BAPTA; and *p* = 0.54 for Control vs. 20 mM BAPTA) (Table 1). Taken together, our data indicate that agonizing I_CaL_ with 25 nM Bay K 8466 does not promote significant AP alternans. However, removing CaT with BAPTA is an alternans promoting intervention.

### 3.4 Slope of AP restitution

Given that the development of AP alternans driven by AP instabilities has been tied to the slope of the APD restitution curve (Nolasco and Dahlen, 1968; Qu and Weiss, 2023), we next plotted Mean-APD_90_ *versus* Mean-DI for each experiment of the Control (Supplementary Figures S3, S4), Bay K 8466 (Supplementary Figure S5), 10 mM BAPTA (Supplementary Figure S6), and 20 mM BAPTA (Supplementary Figure S7) groups. First, it should be noted that the Mean-DI at the ALT-TH was similar between interventions. Specifically, Mean-DI was 47 ± 17 ms in Control, 56 ± 14 ms with 25 nM Bay K 8466 (*p* = 0.21), 49 ± 13 ms with 10 mM BAPTA (*p* = 0.86), and 53 ± 11 ms with 20 mM BAPTA (*p* = 0.40) (Table 1), suggesting that a critical common ionic time-dependent event occurring during the DI may underlie AP alternans formation.

**FIGURE 4:**
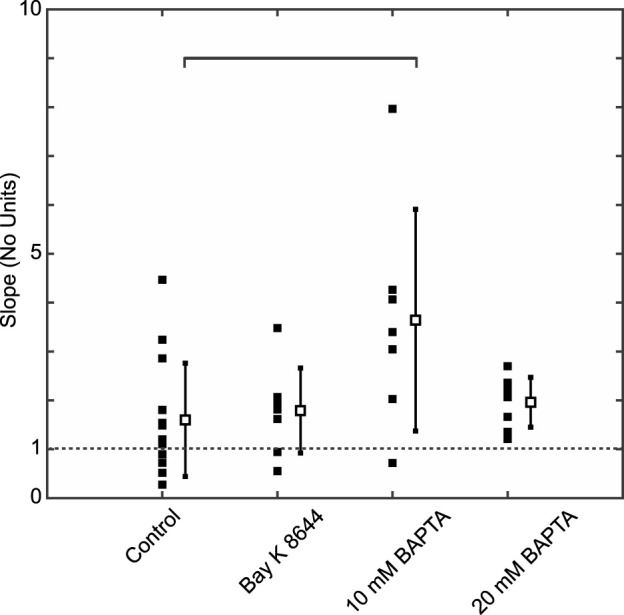
The mean APD restitution curve slope at the ALT-TH is greater than one for all experimental groups. Solid symbols represent individual slope values and the open symbols and error bars the group-wise slope mean and standard deviation values for Control, Bay K 8,644, 10 mM BAPTA, and 20 mM BAPTA groups. Brackets identify comparisons between average values exhibiting significant differences (*p* = 0.012 for 10 mM BAPTA vs. Control, Student’s t-test).

Using a first derivative approach to calculate the restitution slope at the ALT-TH for each cell, we determined that at the onset of alternans the average slope was >1 for all groups (Figure 4). While average values remained similar in the Control, Bay K 8644, and 20 mM BAPTA cells, the slope in cells buffered with 10 mM BAPTA was significantly larger than Controls (Figure 4).

### 3.5 Dynamic response of AP alternans

**FIGURE 5:**
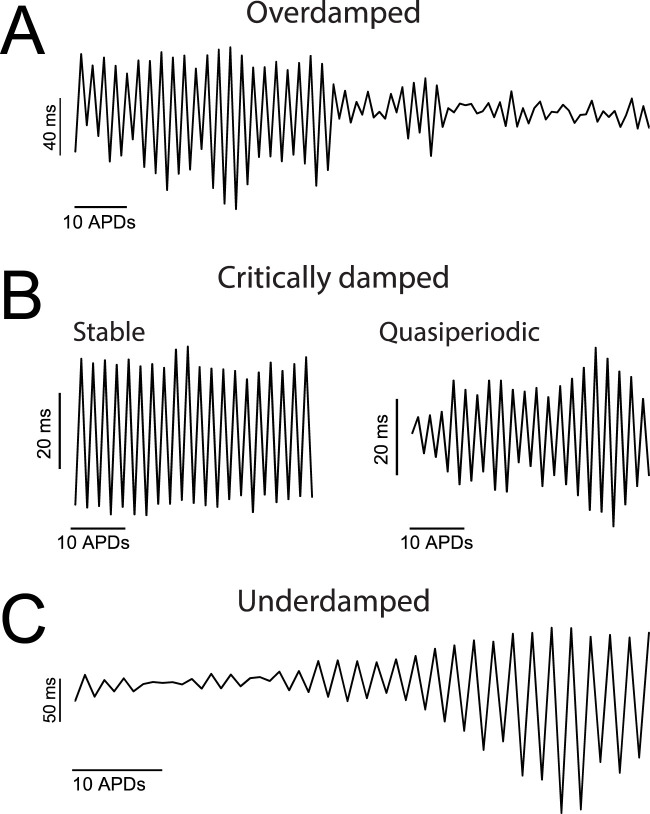
Over extended periods of time AP alternans exhibit heterogeneous dynamic responses. **(A–C)** APD_90_ values plotted against activation number for representative cases of overdamped **(A)**, critically damped **(B)**, and underdamped **(C)** AP alternans. Panel B includes the two categories of critically damped alternans recorded: ‘stable’ and ‘quasiperiodic’.

With current clamping alone, we were able to measure AP alternans for significantly longer periods of time. Inspection of the AP dynamic response to a pacing rhythm change at the scale of tens-to-hundreds of sequential activations revealed that the pattern of developing alternans was diverse (see example in Supplementary Figure S8), in line with previous observations (Dilly and Lab, 1988). For the purpose of visualizing the dynamic response of alternans over extended periods of time we plotted APD_90_ against time during uninterrupted pacing at a fixed PCL (Figure 5). Analysis of such prolonged sequences revealed that alternans could be classified into three major categories: overdamped, critically damped, and underdamped (Figure 5).

Specifically, overdamped alternans were present at a rhythm change, and disappeared (aka resolved) after a variable amount of time. A representative trace of overdamped alternans is shown in Figure 5A. On the other hand, critically damped alternans were either stable (i.e., the alternans magnitude was constant over time) or exhibited lower frequency harmonics (i.e., amplitude modulated aka quasiperiodic) as shown in Figure 5B. Similar quasiperiodic type alternans have been previously described in experimental models (Gilmour et al., 1997) and in simulations (Otani and Gilmour, 1997; Shiferaw et al., 2005). Finally, underdamped alternans were those in which the amplitude of oscillations became progressively larger (Figure 5C), ultimately prompting the conversion of a 2:2 type response (alternans) to a 2:1 type response (a consecutive captured and non-captured beat).

To determine if the formation and pattern of alternans over extended time intervals was modified by the experimental conditions, we classified each AP alternans sequence recorded for every myocyte into one of the above-mentioned categories. Analysis of summary data (Table 2) reveals that AP alternans were more frequently critically damped in alternans positive cells of both the Control (77%) and Bay K 8644 (43.75%) groups. Of note however, the proportion of alternans types in the Bay K 8644 group was significantly altered (*p* = 0.046), with the I_CaL_ agonist lowering the proportion of critically damped alternans while also increasing the proportion of underdamped alternans from 9% to 31.25% (Table 2).

**TABLE 2:** Group wise distribution of critically damped, overdamped, and undamped type alternans.

	Critically damped (%)	Overdamped (%)	Underdamped (%)	Chi-square *p*-value
Control (n = 35)	77	14	9	
Bay K 8644 (n = 15)	43.75	25	31.25	0.046
10 mM BAPTA (n = 12)	41.6	41.6	16.6	0.069
20 mM BAPTA (n = 16)	43.75	12.5	43.75	0.012
10 mM BAPTA + Bay K 8644 (n = 14)	7	14	79	<0.0001

On the other hand, while 10 mM BAPTA myocytes developed a nominally equal proportion of critically damped alternans and overdamped alternans (Table 2), the distribution of alternans types was not significantly different from Controls (*p* = 0.069). Interestingly, overdamped alternans that resolved to non-alternating rhythms during rapid pacing in myocytes perfused with 10 mM BAPTA occurred frequently (5/7 alternans positive myocytes). This finding is consistent with other reports that AP alternans are absent at steady-state pacing in the presence of BAPTA (Goldhaber et al., 2005). Importantly, the AP alternans in 7/8 alternans positive cells treated with 20 mM BAPTA were either critically damped (43.75%) or underdamped (43.75%) (Table 2), and this distribution is significantly different from that of Controls (*p* = 0.012).

### 3.6 Combined CaT buffering and agonizing I_Ca,L_ promotes underdamped alternans

**FIGURE 6:**
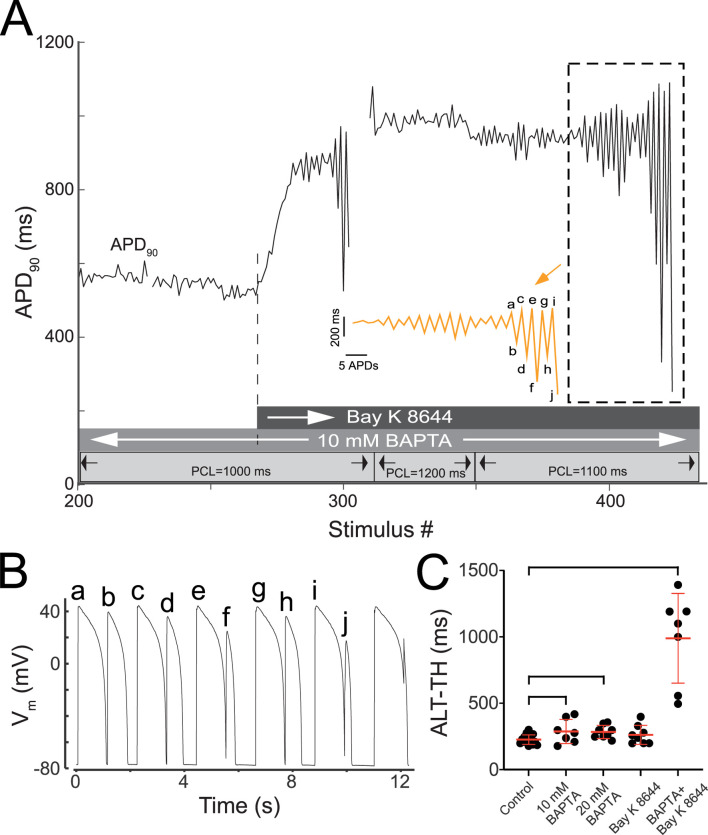
CaT ablation plus Bay K 8644 elicits unrestrained underdamped AP alternans. **(A)** Time-course of APD_90_ changes recorded from a representative CaT buffered myocyte subject to continued pacing (stimulus #200 through #425) during baseline and after perfusion with 25 nM bay K 8644. Three grey horizontal bars overlying the abscissa in panel A provide the timing and duration of relevant experimental interventions. The lightest grey bar shows the intervals at which each PCL was delivered as indicated by the solid vertical lines, the arrows, and the associated labels demarcating each depicted PCL. The intermediate grey bar shows the interval in which 10 mM BAPTA was present. The darkest grey bar shows the interval in which 25 uM Bay K 8644 was present. The initiation of Bay K 8644 delivery is indicated by the white arrow in the darkest grey bar and by the vertically projected dotted line that connects the bar to the APD_90_ data points. The dotted-line box in panel A identifies an episode of underdamped alternans. The adjacent orange trace depicts the APD_90_ sequence inside the dotted box after expanding the *x*-axis and compressing the *y*-axis for improved visualization. Sections containing APD_90_ values associated to 2:1 responses were deleted for clarity. Labels a-to-j identify the final APD_90_ values leading to 2:1 activation. **(B)** V_m_ changes exhibiting the transition from a 2:2 to a 2:1 activation from example identified in the dotted box in panel A. Labels a-to-c trace the correspondence between each AP in panel B and the APD_90_ value in panel A. **(C)** Group-wise comparison of the ALT-TH. Black circles represent the individual ALT-TH values and red lines combined with error bars represent the ALT-TH mean and standard deviation values for Control, Bay K 8644, 10 mM BAPTA, 20 mM BAPTA, and 10 mM BAPTA + Bay K 8644 groups. Brackets identify comparisons between average values exhibiting significant differences (*p* = 0.045, *p* = 0.0065, *p* = 0.00012, respectively 10 mM BAPTA, 20 mM BAPTA, and 10 mM BAPTA plus Bay K 8466 vs. control, Student’s t-test).

As an additional exploration of the feedback loop between AP, CaT, and I_Ca,L_, cells were first loaded with the lowest BAPTA concentration (10 mM) and subsequently perfused with 25 nM Bay K 8466. We hypothesized that the agonizing I_Ca,L_ in the absence of CaT would further increase cellular susceptibility to AP alternans as well as the complexity of those alternans. The results were unexpected, yet still consistent with the hypothesis. Specifically, perfusion of Bay K 8644 upon myocytes with buffered CaT prompted prominent changes in the dynamic response to pacing (Figure 6). During bradycardic constant pacing (1000 ms PCL), AP alternans were not observed with 10 mM BAPTA (Figure 6A), consistent with the lack of alternans observed during normocardic pacing. However, the addition of Bay K 8644 to the CaT free myocyte (Figure 6A, dotted line and darkest grey bar) elicited a fast and prominent increase of APD_90_ (from ∼500 ms at baseline to ∼850 ms) followed by the formation of AP alternans (Figure 6A, stimulus #284–302). Of note, the magnitude of the alternans progressively increased and became unstable. The unrestricted increases in the magnitude of alternans during constant pacing ultimately led to activation block (i.e. 2:1 type response) on stimulus #303. APD_90_ values were not reported during 2:1 pacing. A subsequent attempt to reduce the development of underdamped alternans by increasing the PCL to 1200 ms was successful, but upon readjusting the PCL down towards 1100 ms, an additional episode of underdamped alternans leading to activation block was triggered (Figure 6A dotted box). For better visualization of this transition, the episode of underdamped alternans enclosed in dotted box is replotted with an expanded *x*-axis (orange trace, inset). The final events leading to the activation block in this episode are labeled ‘a’ to ‘j’ in the zoomed-in APD_90_ sequence, and can be traced to the corresponding dynamic transmembrane potential changes depicted in Figure 6B. Note here that the progressive increase in the magnitude of AP alternans leads to the final abortive response to pacing which effectively initiates the 2:1 response sequence (Figure 6B, last unlabeled AP complex and pacing artifact during repolarization). The above-described phenomenon occurred two additional times upon resumption of pacing (not shown). Eventually, by increasing the PCL to 1600 ms, activity devoid of 2:2 type responses was achieved. In all, similar responses to combined 10 mM BAPTA and Bay K 8644 were observed in six additional myocytes. In these myocytes, the proportion of underdamped alternans increased prominently from 9% in control to 79% (*p* < 0.0001) (Table 2). Importantly, the ALT-TH in the CaT buffered myocytes subject to Bay K 8644 was markedly increased to 990 ± 338 ms (*p* < 0.0002 compared to control) (Figure 6C). Taken together, agonizing I_Ca,L_ is not proarrhythmic on its own. Importantly, agonizing I_Ca,L_ in the absence of CaT is proarrhythmic at a cellular level.

### 3.7 CaT and quasiperiodicity

From a theoretical point of view, continuous shifts back and forth from an electromechanically concordant to an electromechanically discordant AP and CaT type relationship have been implicated in the development of quasiperiodic alternans (Shiferaw et al., 2005).

**FIGURE 7:**
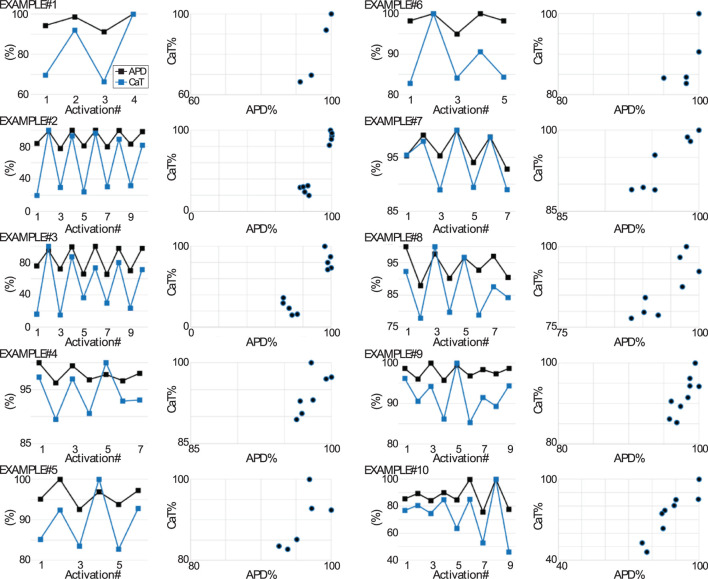
Relationship between AP and CaT during the formation of alternans. Plots depict for 10 tandem AP and CaT recordings (examples #1 through #10) acquired from Control myocytes during the formation of alternans: 1) the simultaneous changes in APD_90_ and CaT amplitude against activation number (leftmost plots for each example) and 2) the APD_90_ *versus* CaT (rightmost scatter plot for each example). The APD_90_ and CaT amplitude data points are expressed as a percentage value, which was determined respectively as the percentage change with respect to the largest APD_90_ value in the recording and as the percentage change with respect to the largest CaT amplitude in the recording. The CaT amplitude for a given CaT in the recording sequence was computed as the CaT’s maximal systolic fluo-4 fluorescence minus the preceding minimal diastolic fluorescence.

See Figure 7 for a quantification of the relationship between APD_90_ and CaT during alternans formation in various examples. Note from the plots in Figure 7 depicting percentage changes of AP and CaT parameters measured on a beat-to-beat basis, that some of the examples (#1, #2, and #3) exhibited a clear demarcation between the two groups of data points forming part of the scatter plots of AP vs. CaT. From a theoretical standpoint (Shiferaw et al., 2005), this distribution is consistent with positive AP-to-Ca^2+^ coupling, negative Ca^2+^-to-AP coupling, and an “instability dominated by voltage”. On the other hand, the scatter plots from a different set of examples (#4, #9, and #10), exhibit a smearing of the distribution of data points in the scatterplot. From a theoretical standpoint (Shiferaw et al., 2005), this type of distribution is partly consistent with shifting dominance in the mechanism (either voltage or Ca^2+^) driving the alternans. Such a shifting dominance scenario has been suggested to underlie the formation of quasiperiodic alternans (Shiferaw et al., 2005). However, a shifting dominance scenario would also require a shift in the AP and CaT relationship from electromechanically concordant to electromechanically discordant, which was not observed. Interestingly, inspection of AP alternans at the scale of tens-to-hundreds of sequential activations, showed that the BAPTA loaded myocytes without a detectable CaT exhibited quasiperiodic oscillations (Supplementary Figure S9; Table 2), indicating that an exclusively sarcolemmal ionic mechanism likely plays an important role in the development of quasiperiodicity.

## 4 Discussion

### 4.1 Main discussion

The purpose of this study was to investigate the ionic basis of voltage driven AP alternans. Based on earlier simulation studies that highlighted I_CaL_ gating as a determinant of AP alternans (Fox et al., 2002), we tested the hypothesis that the CaT dampens action potential alternans. Herein we demonstrate: 1) Ca^2+^ alternans are not required for the development of AP alternans; 2) an AP alternans promoting effect of the Ca^2+^ chelator BAPTA; 3) increasing BAPTA concentrations favor the destabilization of AP alternans; 4) CaT removal in combination with the I_CaL_ agonist and inotropic agent Bay K 8644 can promote the formation of severely underdamped alternans leading to recursive activation block, an arrhythmia triggering event. In short, AP alternans can occur without CaT alternans, the CaT negatively feeds back to attenuate AP alternans via a mechanism involving the I_CaL_. This paradigm is in contrast with a number of studies which suggest that instabilities in the CaT are the cause of AP alternans (Qu and Weiss, 2023; Kulkarni et al., 2019). Importantly, our results are in line with previous computer simulations that identified the I_CaL_ and its sensitivity to changes in intracellular Ca^2+^ as a promoter of steep APD restitution and AP alternans (Fox et al., 2002; Shiferaw et al., 2005). Our study is clinically relevant because it identifies a principle ionic mechanism underlying the formation of voltage driven AP alternans, the cellular driver of T-wave alternans, a known harbinger of ventricular arrhythmia. The protective role of Ca^2+^ may bring about a new paradigm for therapy.

Our results may appear to be in contrast with previous studies demonstrating that CaT removal with BAPTA protected against the development of alternans (Walker et al., 2003; Goldhaber et al., 2005). While the results of both studies are nominally opposite to ours (protective vs. detrimental) for the same concentration of BAPTA (10 mM), we additionally report that 10 mM BAPTA shifted alternans from ‘critically’ to ‘overdamped’ type alternans. Given that AP alternans may resolve over time at this degree of Ca^2+^ chelation, it is possible that the time of measurement affected the results in each of the studies. Partially supporting this hypothesis is a previous observation of transient alternans after a single premature beat and no alternans over much longer pacing durations (Goldhaber et al., 2005). An alternative and complementary hypothesis could be that we used larger step sizes when reducing PCL and did not pace below 180 ms, while others reduced PCL in 5–20 ms decrements achieving, at least in some occasion, final PCLs close to 50 ms (Goldhaber et al., 2005). The different pacing protocols could also affect the temporal formation of alternans as a result of cardiac memory, different rates of intracellular ion accumulation and loss, or other time-dependent electrophysiological properties.

Importantly, when we elevated BAPTA to 20 mM (twice the concentration of previous studies), we made a novel observation that this level of Ca^2+^ buffering promoted underdamped alternans. Under this condition, the applied PCL could not be shortened beyond the ALT-TH. In short, buffering Ca^2+^ appears to shift the complexity of alternans from critically damped, to overdamped, and at increased buffering potency to underdamped.

Aside from the origin of discrepancies amongst studies, our experiments using BAPTA to buffer the CaT demonstrate that Ca^2+^ alternans is not required for the development of rapid-pacing induced AP alternans at physiological temperatures. More importantly, the alternans threshold increased (i.e., slower rate required) when we buffered the CaT, indicating that Ca^2+^ buffering increases the propensity to form alternans. This principle finding must be viewed in tandem with earlier studies demonstrating the close to reverse scenario, where Ca^2+^ alternans can form during an alternans-free AP-clamp (Chudin et al., 1999). Others, using a similar approach, showed that CaT could develop both electromechanically concordant or discordant alternans, in a manner which was independent of AP dynamics (Kanaporis and Blatter, 2015). Taken together, the data indicate that both signals are capable of generating alternans inherently when voltage and Ca^2+^ are uncoupled.

Another method to determine whether AP or CaT are the primary driver of alternans would be to compare the ALT-TH of each signal. Our data place the AP alternans threshold of the BAPTA loaded myocyte prominently higher (∼280 ms) than that of the unclamped AP which is ∼220 ms, in accordance with previous studies (Mahajan et al., 2008). To the best of our knowledge, the equivalent measure for the CaT, the inherent Ca^2+^ alternans threshold, has not been experimentally determined in rabbit ventricular myocytes at physiological temperatures. In canine myocytes the alternans-free AP-clamped threshold for Ca^2+^ alternans was ∼258 ms (Wilson et al., 2009), which is still lower than the ∼280 ms AP alternans threshold in the BAPTA loaded myocytes of our study. Side-by-side comparison of the available information on the inherent thresholds for the AP and the CaT signals suggests that AP alternans occur at higher ALT-TH. Given the above, and recognizing the limitations of interstudy data comparisons, our data is consistent with a model in which rapid-pacing induced AP alternans is governed by a voltage driven instability which is dampened by intracellular Ca^2+^ cycling. In line with this notion, our dual AP and CaT recordings always exhibited a relationship between the signals such that long/short APD was associated with a large/small CaT, which is expected for a graded type Ca^2+^ release triggered by voltage. This type of interaction between the signals has been previously denominated a ‘positive voltage to calcium type coupling’ (Qu and Weiss, 2023).

Given that BAPTA prolonged APD both at baseline and at the ALT-TH, it is likely that the intracellular Ca^2+^ mediated -inactivation of I_CaL_ is the signal responsible for dampening the AP alternans. In line with this, simulations by Fox et al. showed that the magnitude and incidence of alternans could be altered by varying the time constant for Ca^2+^ induced inactivation of I_CaL_ (Fox et al., 2002). In addition, our observation that the higher BAPTA concentration promotes underdamped alternans indicates that the extent of Ca^2+^ buffering is an important determinant of alternans stability. Specifically, we propose that this observation may be tied to the local regulation of I_CaL_ by calmodulin and changes in Ca^2+^ concentration which take place very near or at the channel pore (Christel and Lee, 2012). Supporting the notion that changing BAPTA concentration from the 10 mM–20 mM may influence the formation of alternans via a change in the local regulation of I_CaL_, previous studies reported that buffering provided by 10 mM BAPTA reduces, but does not abolish CaT induced inactivation of I_CaL_ (Sham, 1997; Dick et al., 2008).

In considering a voltage driven mechanism of alternans, the specific ion channels generating the instability that cause AP alternans remain to be further delineated. Of note, our data show that changes in the ALT-TH closely tracked the changes in APD. Given that changes in the APD are tied to reciprocal changes in the DI, the finding supports the presence of an ionic time-dependent event occurring during the DI as the cause of AP alternans. Indeed, the finding that values of DI measured at the ALT-TH was similar across experimental groups exhibiting distinct APD values at ALT-TH support this. Additionally, the fact that cells with intact CaT and cells loaded with BAPTA had similar DI values at ALT-TH suggests that the voltage instability causing alternans may be less dependent on the presence or absence of Ca^2+^. Of note also, the marked alternans promoting effect that the I_CaL_ agonist Bay K 8644 had on the BAPTA loaded myocytes indicates that the voltage and time-dependent properties of I_CaL_ may play a key role in the formation of alternans.

**FIGURE 8:**
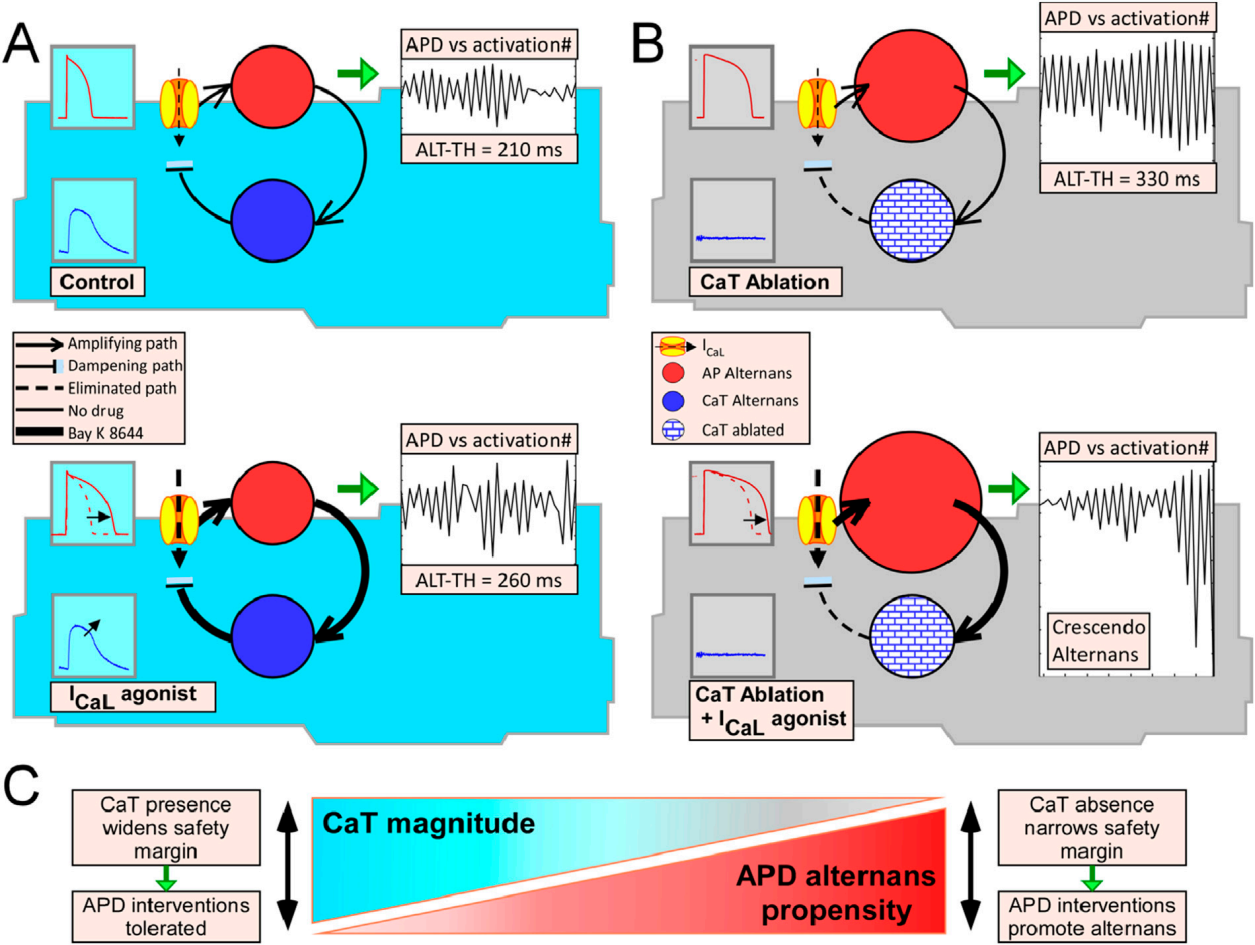
Diagram of the mechanistic paradigm in which voltage driven AP alternans and CaT are linked via a negative feedback loop. Voltage driven AP alternans is represented by a solid red circle in which the diameter is proportional (qualitatively only) to the ALT-TH. The solid blue circle represents the CaT oscillations which are driven by the AP changes (amplifying path represented by a continuous arrow going from red-to-blue circles) and simultaneously provide a negative feedback cue upon the oscillating APs via Ca^2+^ dependent inactivation of I_CaL_ (dampening path represented by a continuous line-and-block symbol going from blue circle to the ion channel). **(A)** Blue myocytes represent cells with a conserved CaT. In this scenario, AP alternans is driven by a closed uninterrupted feedback loop, in which the amplifying path and the dampening path counteract each other, both in Control (upper cell) and when I_CaL_ is agonized (lower cell). In the latter scenario, agonizing I_CaL_ leads to increases of both the amplifying path and feedback path (lines symbolizing the paths are thicker) and therefore the net result is no change on the ALT-TH (red circle remains of similar diameter), which is analogous to our experimental observations with Bay K 8644. **(B)** Grey myocytes represent cells lacking a CaT. In this scenario, removing CaT (patterned blue circles) effectively interrupts the feedback loop by eliminating the dampening cue (dotted line and block symbol indicate eliminated path), which leads to a disproportionate development of AP alternans (i.e., the ALT-TH becomes larger symbolized by the larger solid red circle). By this mechanism, CaT ablation alone (upper cell) can promote the formation of AP alternans as indeed was observed experimentally with the Ca^2+^ buffer BAPTA. Agonizing the I_CaL_ in this scenario (lower cell), has a profound effect leading to the recurrent formation of underdamped alternans (largest red circle) because the drug induced increase in the amplifying path (line symbolizing the path is thicker) does not have a dampening counterpart, which is analogous to our experimental observations when combining BAPTA and Bay K 8644. **(C)** According to the paradigm described, decreasing the CaT amplitude (rightmost tip of blue triangle) increases the propensity of AP alternans (rightmost base of red triangle), and *vice versa* (leftmost section of both triangles). Thus, the presence of a conserved CaT widens the safety margin for interventions targeting the AP or CaT, whereas CaT absence (or reduction) narrows the safety margin effectively increasing the propensity to induced AP alternans when interventions targeting AP or CaT are delivered.

Figure 8 depicts schematically the proposed AP alternans dampening mechanism involving a negative feedback loop connecting the membrane and intracellular Ca^2+^ signals, for the four experimental conditions that we tested. Under this alternans-driven-by AP scheme, Ca^2+^ does not directly cause alternans but plays a critical role in the reduction and stabilization of the membrane oscillations. Following this mechanistic reasoning, myocytes with conserved CaT exposed to Bay K 8644 would be expected to exhibit a modest change in the formation of alternans, as indeed we and others observed (Saitoh et al., 1988). By the same reasoning, the absence of a dampening cue in the CaT buffered myocytes subject to Bay K 8644 underlies the dramatic formation of underdamped alternans that effectively increased the ALT-TH by fourfold in this group of cells. Given the scenario proposed in Figure 8, we further speculate that the determinants of intracellular Ca^2+^ release restitution (Cely-Ortiz et al., 2020) are key in modulating AP alternans through dynamic dampening of the AP oscillations. Experiments designed to measure the inherent capability of CaT to develop alternans at physiological temperatures as well as tandem measurements during transition to alternans will be required to further clarify the mechanism driving AP alternans. Towards this, continuous tandem recordings of Vm and CaT during the transition to alternans carried out at room temperature showed that the onset of APD alternans coincided in time with the onset of CaT alternans (Kanaporis and Blatter, 2015). In contrast, simultaneous optical recordings of AP and CaT during rapid pacing induced alternans in intact rabbit hearts showed a temporal hierarchy in the development of alternans with CaT amplitude alternans occurring before APD alternans (Visweswaran et al., 2013). Others, however, reported the simultaneous initiation of both APD and CaT alternans in dog hearts (Wilson et al., 2009). Given the temporal and spatial limitations of optical mapping, particularly the inability to resolve where in 2-dimensional tissue a phenomenon starts, isolated myocytes may be useful towards determining if any of the signals is the alternans precursor.

Towards determining how removing the CaT affects the formation of alternans, others used either caffeine (Kihara and Morgan, 1991; Saitoh et al., 1989; Hirayama et al., 1993), ryanodine (Kihara and Morgan, 1991; Saitoh et al., 1989; Kanaporis and Blatter, 2015), thapsigargin (Pearman et al., 2018), or ryanodine in combination with thapsigargin (Goldhaber et al., 2005). All listed approaches, which were reported as alternans protective, hinge on preventing sarcoplasmic reticulum Ca^2+^ accumulation, which should markedly increase cellular free Ca^2+^ in contrast to the buffering effect of BAPTA. The contrasting conclusion of whether inhibiting Ca^2+^ cycling is pro and anti-alternans may therefore be dependent on the method to attenuate CaT. It is plausible that differing free Ca^2+^ levels between approaches may result in distinct effects on the Ca^2+^ -dependent inactivation of I_CaL_, with approaches increasing the cytosolic Ca^2+^ levels promoting I_CaL_ inactivation (protective) *versus* those approaches reducing cytosolic Ca^2+^ levels preventing I_CaL_ inactivation (detrimental). Additional studies designed to test how a range of cytosolic Ca^2+^ concentrations alter AP alternans could be insightful.

#### 4.2 Clinical implications

The notion that CaT provides an alternans protective effect via a bi-directional coupling mediated dampening mechanism has practical implications related to the delivery of therapy since various cardiac conditions are associated to reduced CaT. A case in point is heart failure, which has been associated with reductions of the CaT of up to 50% (Piacentino et al., 2003) or more (Beuckelmann and Erdmann, 1992), as well as to an increased alternans propensity (Wilson et al., 2009). In this scenario, it is plausible that reduced CaT may less efficiently ‘dampen’ (voltage-driven) AP alternans thus effectively promoting their development in the diseased heart. According to our data, this could be particularly damaging if therapy involved activating I_CaL_. However, whether partial reduction of the CaT is pro-AP alternans during heart failure remains to be investigated.

#### 4.3 Limitations of our study

Our data was collected from isolated myocytes that lack electrical coupling between myocytes which may theoretically impact the development of alternans. Nonetheless, previous studies suggest that electrical coupling between myocytes does not have an effect on the ionic processes determining alternans, but rather impacts the spatial distribution of alternans in the whole heart by altering the threshold for the formation of spatially discordant alternans (Kjolbye et al., 2008). An additional limitation related to our approach, is the discrete nature of CaT recordings which precluded us from simultaneously measuring AP and CaT during the transition to alternans due to the uncertainty of this event. Continuous tandem recordings of both AP and CaT will be important to determine the time of onset of AP alternans *versus* CaT alternans. We should also point out that the proposed Ca^2+^ dampens AP alternans paradigm as described herein is applicable to alternans developing in healthy hearts in response to rapid pacing. Future studies will be required to determine the nature of the interaction between AP instabilities and Ca^2+^ instabilities in diseased hearts. In addition, it was shown that it is possible to modify the type of coupling between the CaT and the AP (i.e., positive vs. negative coupling) by applying interventions targeting either the sodium-calcium exchanger or the I_CaL_ (Wan et al., 2012). Given this, caution should be exerted when translating the conclusions of our study to hearts in which pharmacological (or other) interventions may have modified the mechanism driving AP alternans. Regarding the proposed Ca^2+^ dampens AP alternans paradigm it is also important to point out that our argumentation in favor of this paradigm partly relies on interstudy comparisons of the alternans thresholds of the uncoupled AP and the uncoupled CaT signals. An additional limitation relates to the fact that the experimental conditions may modify the cytoplasmic composition, in particular the concentration and/or distribution of the physiological Ca^2+^ buffers. Given that Ca^2+^ buffers may be fixed or mobile (Eisner et al., 2023), it is possible that during whole-cell patch clamp recordings the mobile Ca^2+^ buffers are washed-out rendering the control conditions of the patch clamp experiments different from the normal heart. Finally, the classification of alternans into critically damped, overdamped, and underdamped types implemented in this study was based on a qualitative description of alternans patterns developing over ten-to-hundreds of activations, using an approach similar to that reported by Dilly and Lab (1988). A more quantitative assessment of how two coupled oscillators interact in the presence of a negative feedback loop by means of computational or mathematical models of AP and CaT is envisioned for future studies.

## Data Availability

The raw data supporting the conclusions of this article will be made available by the authors, without undue reservation.
