# Supplementary material for: The calcium transient coupled to the L-type calcium current attenuates cardiac alternans
Source: Front Physiol. 2024 Sep 27;15:1404886. doi: 10.3389/fphys.2024.1404886 (PMC11466891; doi:10.3389/fphys.2024.1404886)
Supplement: Supplementary file 1 [file DataSheet1.pdf]

Supplementary Material

The Calcium Transient Coupled to the L-Type Calcium Current Attenuates Cardiac Alternans

Mark Warren[1]*, Steven Poelzing[1,2,3]

*** Correspondence:** markwarren@vt.edu

1 Supplementary materials and methods

Myocyte Isolation and experimental preparation

All procedures involving animals were approved by the Animal Care and Use Committee of the University of Utah and complied with the American Physiological Society's *Guiding Principles in the Care and Use of Animals.* For isolation of rabbit ventricular myocytes, adult rabbits (1.5-1.7 Kg) were anesthetized with sodium pentobarbital (50mg/kg, i.p.) and the excised heart perfused with solutions gassed with 100% O_2 and held at 37°C, pH 7.3.[1] Myocytes were isolated from the hearts by combining enzymatic tissue digestion using 0.15 mg/ml collagenase P (Roche Diagnostic, Manheim, Germany) plus 0.05 mg/ml protease (type XIV, Sigma Chemical, St. Louis, MO, USA) and mechanical trituration as described.[1] Rod-shaped myocytes with well-defined striations were placed in a 1-ml flow-through chamber mounted on the stage of an inverted microscope (see below for more details) and were superperfused with a *N*-2-hydroxyethylpiperazine-*N*'-2-ethanesulfonic acid (HEPES)-buffered Tyrode solution containing (in mM) 126.0 NaCl, 4.4 KCl, 1.0 $MgCl_2$, 1.1 $CaCl_2$, 11.0 dextrose, and 24.0 HEPES (free acid) titrated to pH 7.40 with NaOH. Where indicated in the text the cell bathing solution contained 25 nM of the L-type Ca2+ channel opener Bay K 8644 (Tocris Bioscience, UK) diluted into

the superfusate. Bathing solutions flowing continuously through the bath at approximately 4-5 ml/min were maintained at 36.5±1.0 °C.

Pipette filling solutions

The normal pipette filling solution used for recording the transmembrane voltage (V_m) (**Supplement Fig. 1**, black traces) contained (in mM): 110.0 KCl, 5.0 NaCl, 5.0 MgATP, 5.0 phosphocreatine, 1.0 NaGTP, and 10.0 HEPES, titrated to pH 7.2 with 1 M KOH. Where indicated, the pipette solution also contained either 10.0 mM or 20.0 mM BAPTA (1,2-Bis(2-aminophenoxy)ethane-N,N,N',N'-tetraacetic acid; Tocris Bioscience, UK) used to effectively buffer the Ca^{2+} transient (CaT)(**Fig. 2** and **Supplement Fig. 1**, blue traces).

Cellular electrophysiology measurements, pacing protocols, and experimental groups

The V_m was measured using a suction whole cell ruptured patch borosilicate glass pipette (2-4 MΩ tip) and an Axoclamp 2B amplifier (Molecular Devices, Ca) in bridge mode as previously described.[2] The V_m was recorded continuously at a sampling rate of 50 kHz by means of an acquisition system (AxoScope software; Molecular Devices, Ca). Action potential (APs) were initiated using a stimulator (Crescent Electronics, Utah) to inject an intracellular rectangular shaped depolarizing current pulse (duration 3-4 ms). In order to minimize the influence of the stimulus on the recorded APs, the amplitude of the stimulating current was set so that the stimulus artifact in the recorded V_m signals could be resolved from the upstroke of the AP. In meeting this criterion, the

resulting depolarization current amplitude was 1.1-1.4x that of the threshold current. In two (2/22) control myocytes, the amplitude of the stimulating current was set to 2x that of the threshold current.

Measurement of intracellular Ca^{2+} changes

The fluorescent probe fluo-4 was used to monitor changes in intracellular Ca^{2+} ($[Ca^{2+}]_i$) by means of an epifluorescence imaging set up built in tandem with the cell electrophysiology system. To load the dye, myocytes were incubated in normal control solution containing 10 μM fluo-4 AM (Invitrogen) and 0.3 mM probenecid for 20-25 minutes at 30 ^oC as previously described.[1,3] Probenecid (0.3 mM) was included in the bathing solutions to slow down the washout of fluo-4 from the cell interior.

Once the stained myocytes were placed in the chamber, the fluorophore was excited using a 485 nm light source coupled to the microscope stage via a dichroic mirror. The emitted fluo-4 fluorescence was collected using a 40x objective lens (Nikon Fluor, NA=1.30 Oil immersion) and the travelling beam filtered (532 nm, band-pass filter) and projected, via the side-port of the microscope, upon an attached EMCCD camera (iXon 860, Andor Technology, Belfast, UK) configured to record images at a resolution of 64x64 pixels and 860 frames/s. Additionally, to reduce the amount of dye photo bleaching and photo-toxicity we limited exposure of myocytes to the excitation beam using a system of shutters synchronized to the EMCCD camera acquisition and to the pacing pulse train as previously described.[4] Typically, the shutter system was configured to record 2-second-long movies of fluo-4 fluorescence at the test pacing cycle length (PCL) of interest, and the exact recording timing registered to precisely time-align the V_m and $[Ca^{2+}]_i$ signals.

To obtain a measure of $[Ca^{2+}]_i$ changes including the CaT, movies of fluo-4 fluorescence were masked to select pixels overlying the myocyte imaged area. The fluorescent signals from the selected pixels were then averaged in order to construct a spatial averaged representation of the fluo-4 fluorescent changes occurring within the cell. **Supplement Fig. 1** shows simultaneous time-aligned recordings of AP and CaT obtained from a cell constantly paced at a cycle length of 1000 ms during 20 minutes using the above-described techniques. Note that for each time point the AP and CaT shapes remain almost unchanged, although a sizable parallel reduction in the systolic and diastolic fluo-4 fluorescence levels were observed. All experiments described herein were done well within this time frame. **Figure 2** depicts simultaneous recordings of AP and $[Ca^{2+}]_i$ and demonstrates that the BAPTA concentrations we used effectively buffered the CaT below detection level in all experimental conditions used throughout the study. None of the cells loaded with BAPTA contracted while being paced.

Measures of AP and detection of AP duration (APD) alternans

For each recorded AP in response to the pacing protocol depicted in **Supplement Fig. 2** we determined the following parameters: 1) the temporal derivative of the time dependent V_m (dV_m/dt); 2) the maximum value attained by the dV_m/dt (dV_m/dt_{max}); 3) the activation time, which we defined as the time at which dV_m/dt_{max} occurred; 4) the resting membrane potential (V_r), which we defined as the value of V_m just prior to the depolarizing pulse used to activate the cell; 5) the AP amplitude (APA), which we defined as the difference between the V_r and the maximum value of V_m achieved during cellular activation; 6) the action potential duration (APD) at 90% repolarization (APD_{90}), which we defined as the time interval between the activation time and the time at which the

repolarizing V_m attained 90% of APA; 7) the diastolic interval (DI), defined as the time interval between the time of 90% repolarization of the preceding AP and the activation time of the AP in consideration.

1. N. Saegusa, V. Garg, K.W. Spitzer. Modulation of ventricular transient outward K(+) current by acidosis and its effects on excitation-contraction coupling. *Am J Physiol Heart Circ Physiol*. 2013;304:H1680-1696. doi: 10.1152/ajpheart.00070.2013
2. M. Zaniboni, A.E. Pollard, L. Yang, K.W. Spitzer. Beat-to-beat repolarization variability in ventricular myocytes and its suppression by electrical coupling. *Am J Physiol Heart Circ Physiol*. 2000;278:H677-687. doi: 10.1152/ajpheart.2000.278.3.H677
3. N. Saegusa, E. Moorhouse, R.D. Vaughan-Jones, K.W. Spitzer. Influence of pH on Ca(2)(+) current and its control of electrical and Ca(2)(+) signaling in ventricular myocytes. *J Gen Physiol*. 2011;138:537-559. doi: 10.1085/jgp.201110658
4. M. Warren, K.W. Spitzer, B.W. Steadman, T.D. Rees, P. Venable, T. Taylor, J. Shibayama, P. Yan, J.P. Wuskell, L.M. Loew, A.V. Zaitsev. High-precision recording of the action potential in isolated cardiomyocytes using the near-infrared fluorescent dye di-4-ANBDQBS. *Am J Physiol Heart Circ Physiol*. 2010;299:H1271-1281. doi: 10.1152/ajpheart.00248.2010

2 Supplementary Figures

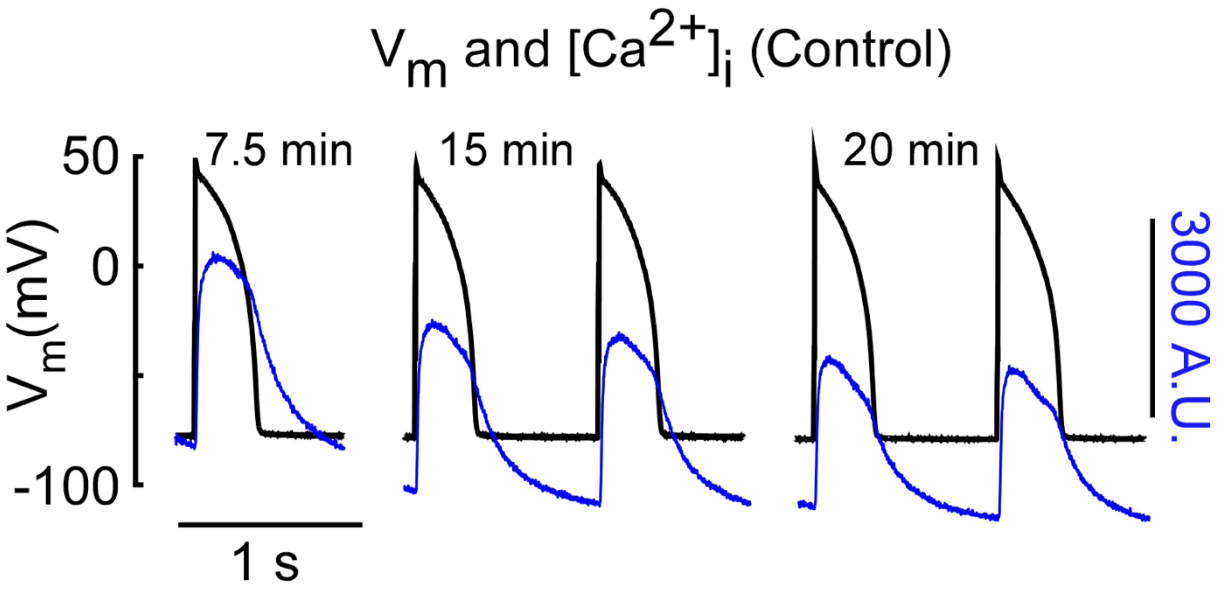

Supplementary Figure 1

Supplementary Figure 1. Measurement of V_m and $[Ca^{2+}]_i$ in isolated myocytes. **A.** Simultaneous recordings of AP and CaT obtained from a constantly paced (PCL=1000 ms) myocyte patched with normal pipette solution and superfused with normal Tyrode solution. The labels indicate the time of recording after attachment of the glass pipette.

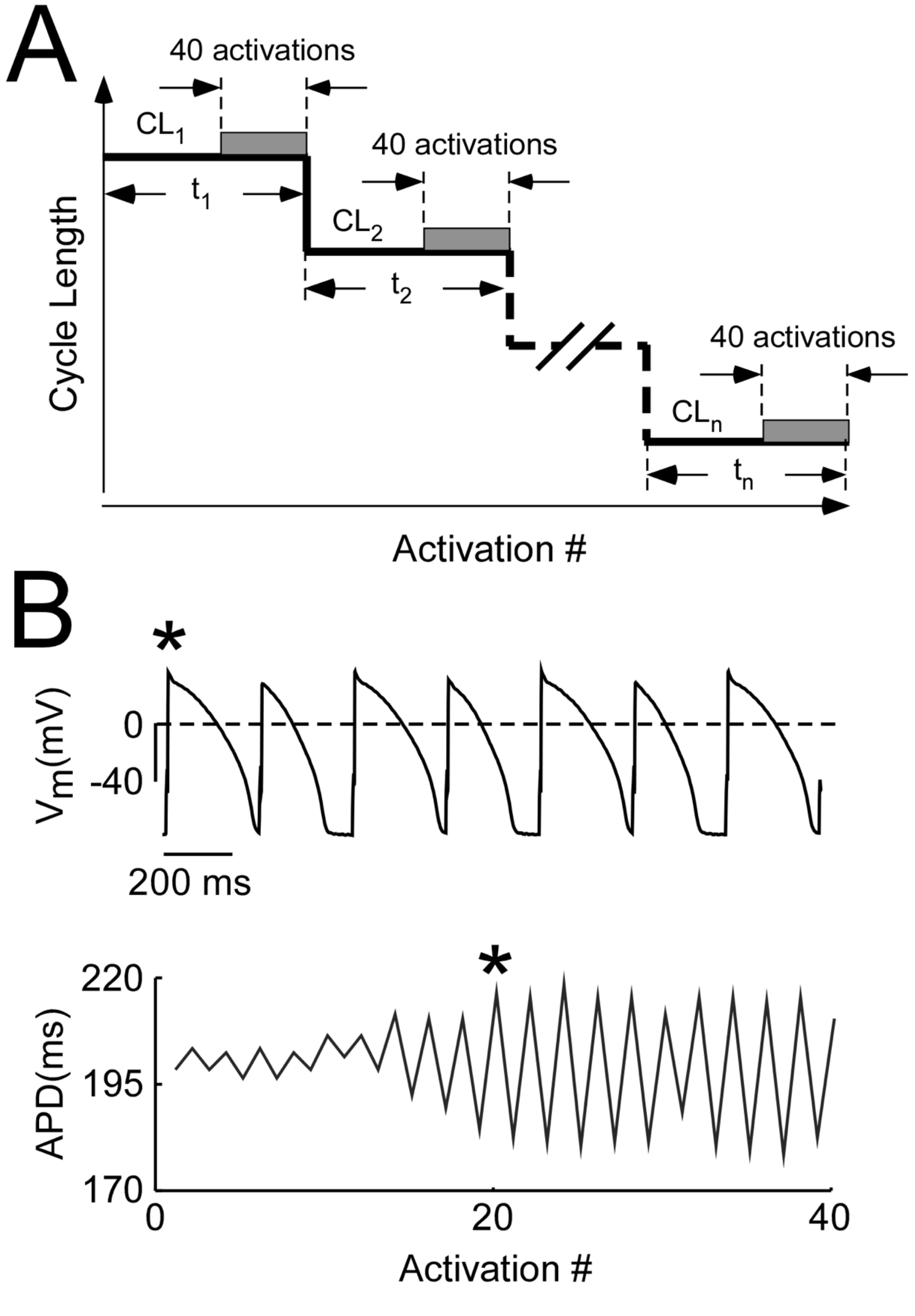

Supplementary Figure 2

Supplementary Figure 2. Methods for generating and detecting AP alternans. **A.** Sketch depicting a generic pacing protocol applied to myocytes. The labels CL_i (i=1 to n) identify the series of shortening PCL values applied to the myocyte. The label t_i (i=1 to n) indicates the variable time (30-90 s) elapsing at each test PCL. The grey boxes represent the 'analysis window', that is the last 40 activations recorded at each test PCL. **B.** (Upper trace) V_m recording depicting typical APs during a 2:2 type response induced by rapid pacing. (Lower trace) The sequence of 40 APD_{90} values conforming the 'analysis window' from which the APs above were selected. The asterisk indicates the correspondence between the first depicted AP and the APD_{90} measure.

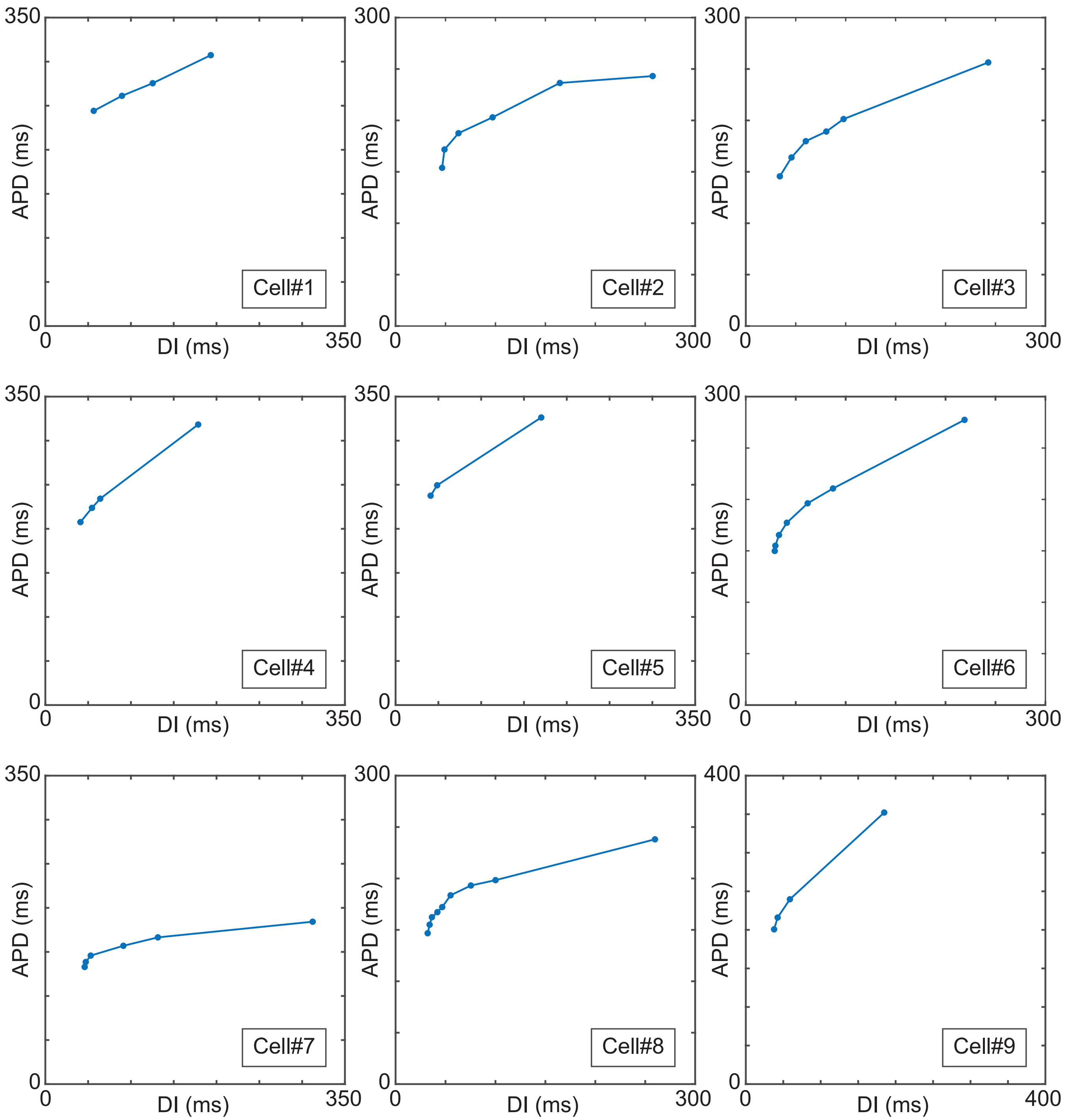

Supplementary Figure 3

Supplementary Figure 3. APD restitution curves for Control. Each panel depicts the Mean-APD_{90} vs Mean-DI plots and interpolated curves for individual alternans positive control myocytes. The figure depicts plots for 9 out of 14 such myocytes (plots for myocytes 10 through 14 are shown in **Supplementary Figure 4**). Plotted points correspond to measures obtained during cellular pacing at PCL values ranging from 500 ms to the shortest PCL applied.

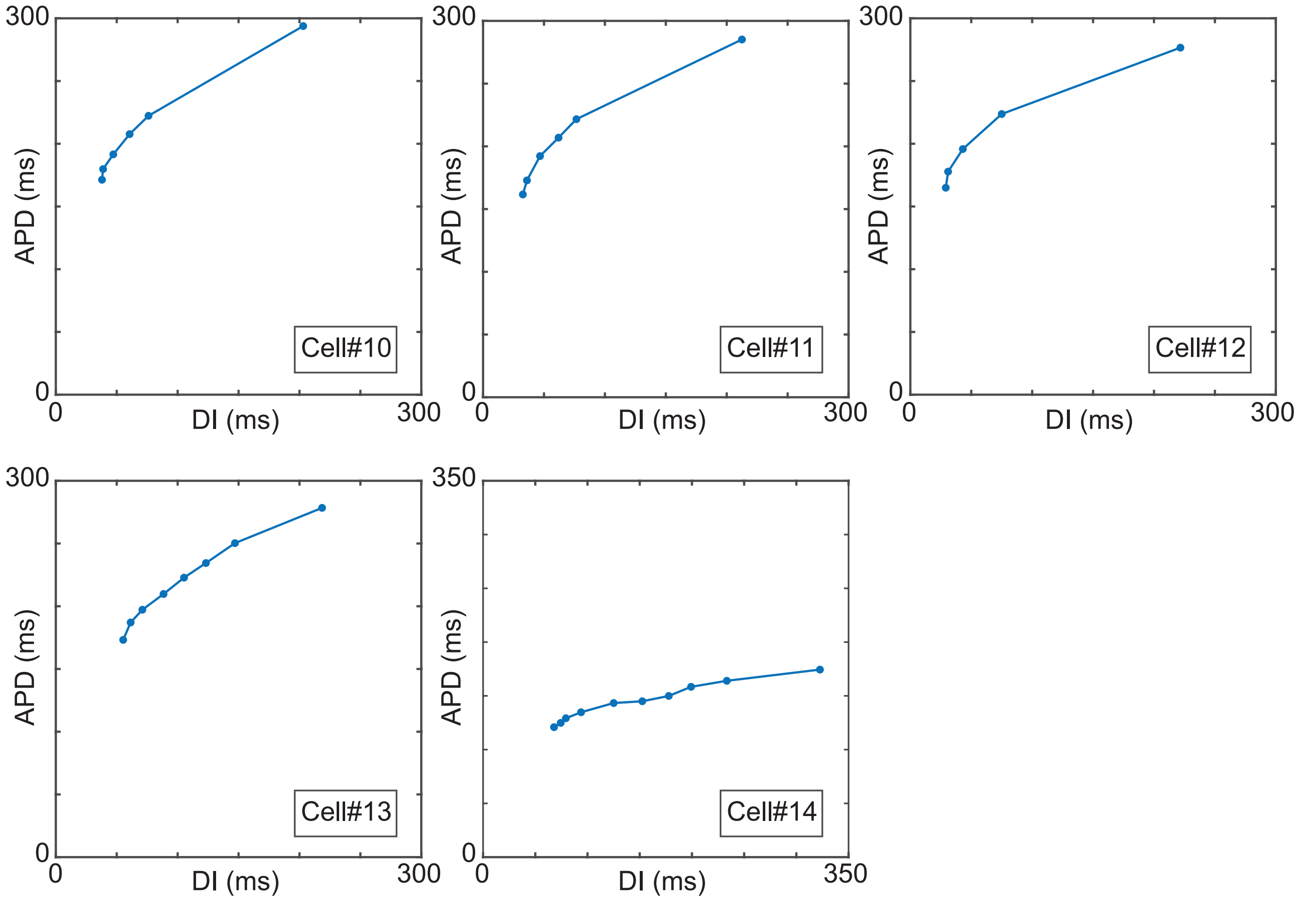

Supplementary Figure 4

Supplementary Figure 4. APD restitution curves for Control. Each panel depicts the Mean-APD_{90} vs Mean-DI plots and interpolated curves for individual alternans positive control myocytes. The figure depicts plots for additional 5 out of 14 alternans positive control myocytes not shown in **Supplementary Figure 3**. Plotted points correspond to measures obtained during cellular pacing at PCL values ranging from 500 ms to the shortest PCL applied.

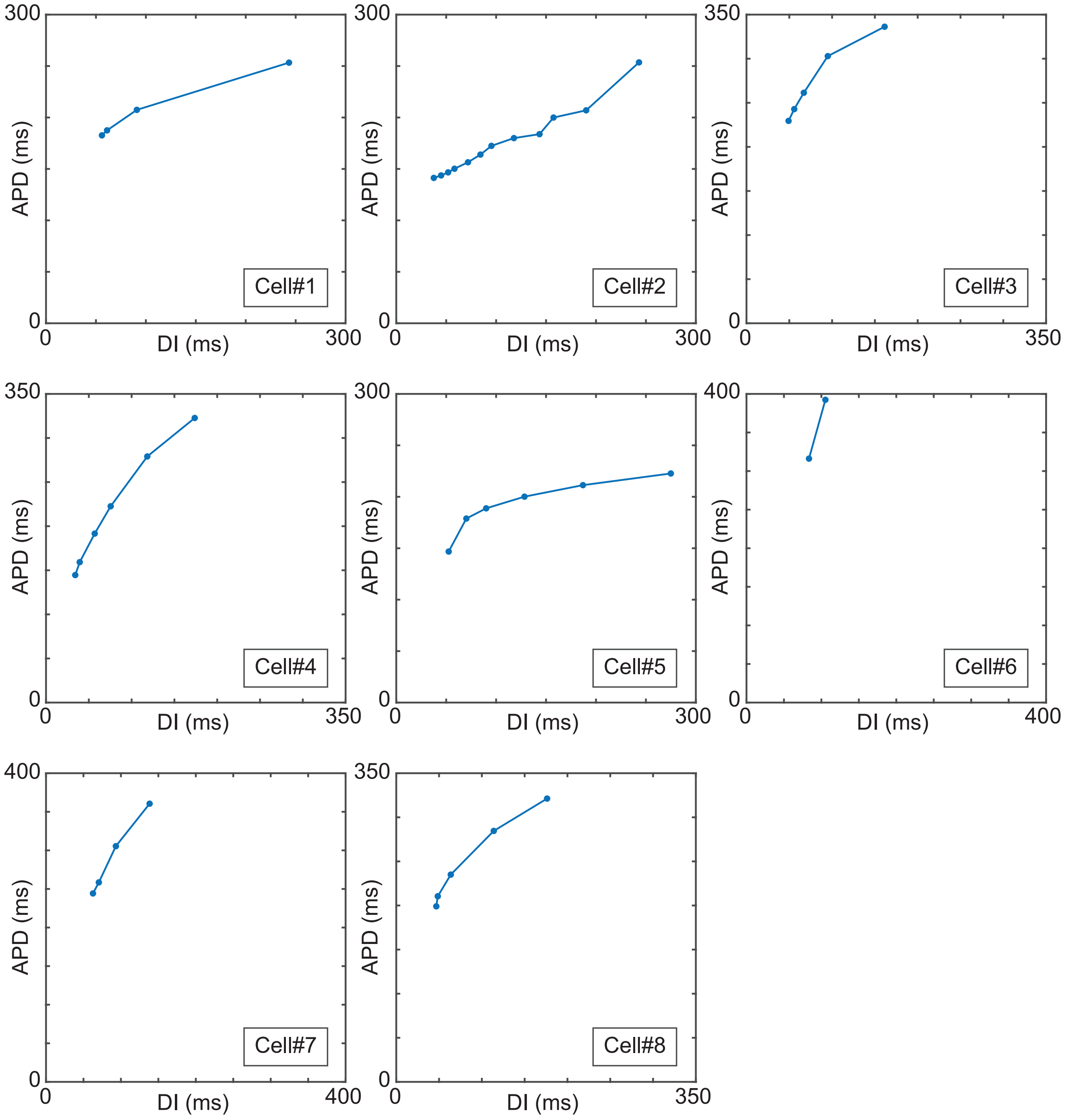

Supplementary Figure 5

Supplementary Figure 5. APD restitution curves for Bay K 8644. Each panel depicts the Mean-APD_{90} vs Mean-DI plots and interpolated curves for individual alternans positive Bay K 8644 myocytes. Plotted points correspond to measures obtained during cellular pacing at PCL values ranging from 500 ms to the shortest PCL applied.

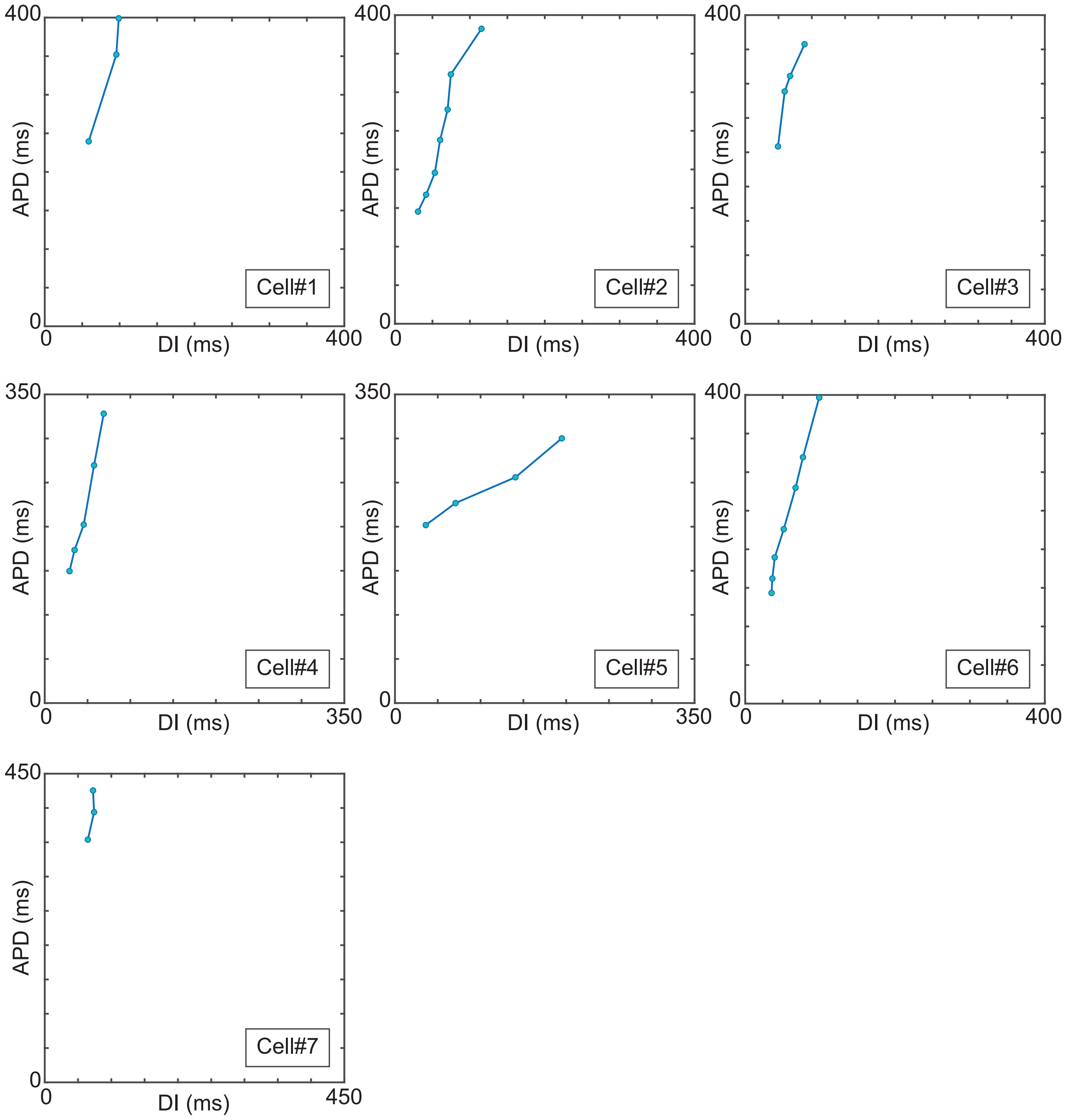

Supplementary Figure 6

Supplementary Figure 6. APD restitution curves for 10 mM BAPTA. Each panel depicts the Mean-APD_{90} vs Mean-DI plots and interpolated curves for individual alternans positive 10 mM BAPTA myocytes. Plotted points correspond to measures obtained during cellular pacing at PCL values ranging from 500 ms to the shortest PCL applied.

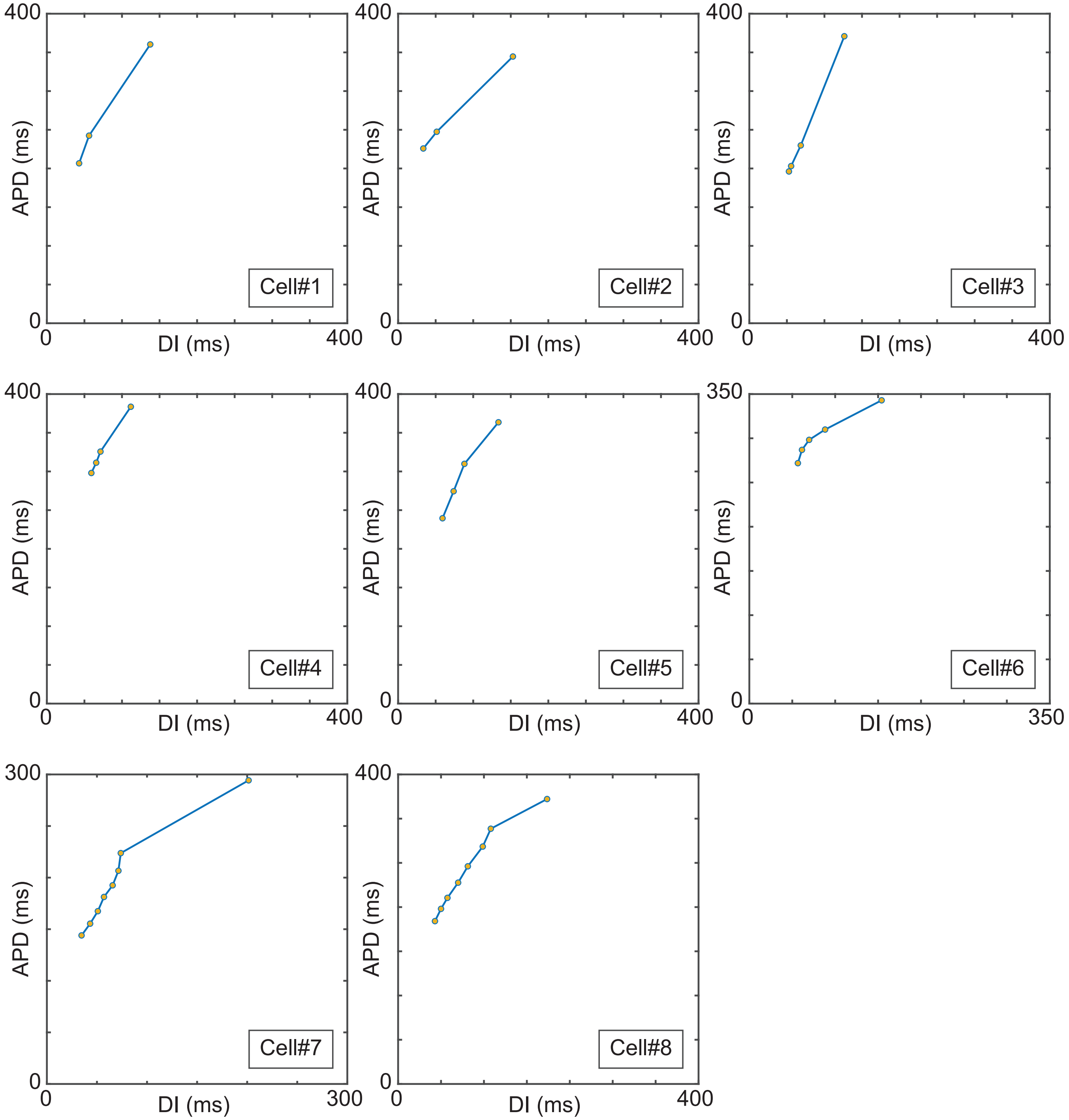

Supplementary Figure 7

Supplementary Figure 7. APD restitution curves for 20 mM BAPTA. Each panel depicts the Mean-APD_{90} vs Mean-DI plots and interpolated curves for individual alternans positive 20 mM BAPTA myocytes. Plotted points correspond to measures obtained during cellular pacing at PCL values ranging from 500 ms to the shortest PCL applied.

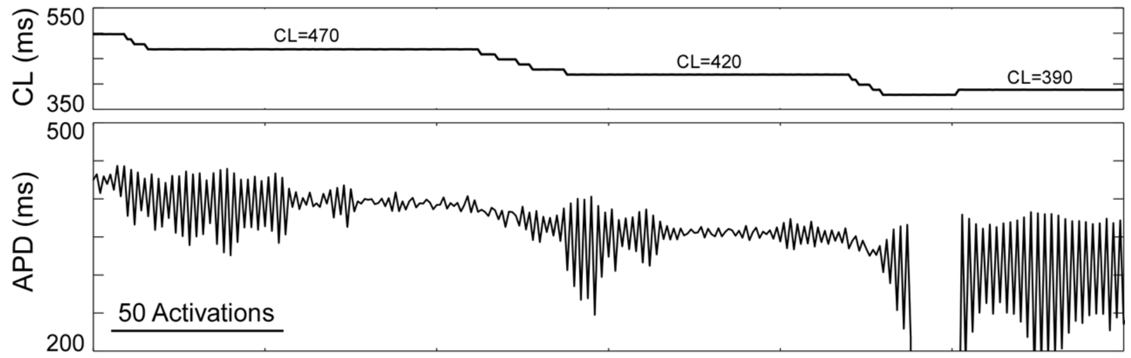

Supplementary Figure 8

Supplementary Figure 8. Time course of APD_{90} values in response to pacing over extended periods of time in a typical myocyte. The figure shows for a typical myocyte the applied PCL against activation number (upper panel) and the recorded APD_{90} values against activation number (lower panel). The depicted measures highlight how recordings from a single cell resulted in a heterogeneous response to pacing that included overdamped, critically damped, and underdamped alternans. The section of the APD_{90} trace with no values corresponds to intermittent capture (2:1 type response) which precluded the correct calculation of APD_{90} for the corresponding activations. Labels in the upper panel indicate the PCL value during sections of the pacing protocol where PCL remained constant for an extended time.

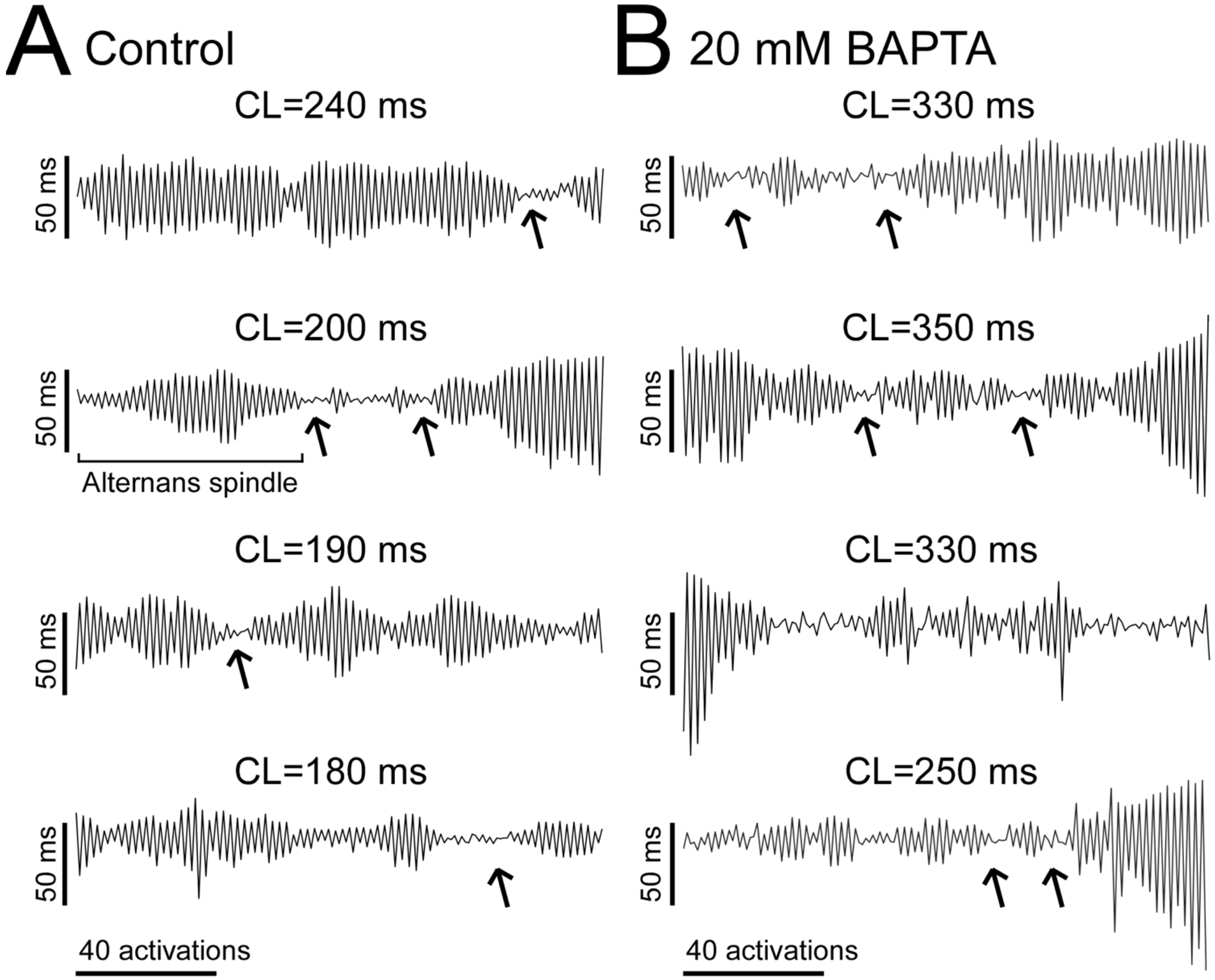

Supplementary Figure 9

Supplementary Figure 9. **Quasiperiodic APD oscillations in myocytes with conserved CaT (Control) and in myocytes with buffered CaT (20 mM BAPTA).** Time course of APD_{90} values corresponding to 150 consecutive activations of constantly paced cells at PCLs that elicited alternans in four control (**A**) and four 20 mM BAPTA (**B**) myocytes. The arrows identify some of the 'nodes' joining distinct quasiperiodic APD_{90} oscillations.
